# Methods and Challenges in Shot Boundary Detection: A Review

**DOI:** 10.3390/e20040214

**Published:** 2018-03-23

**Authors:** Sadiq H. Abdulhussain, Abd Rahman Ramli, M. Iqbal Saripan, Basheera M. Mahmmod, Syed Abdul Rahman Al-Haddad, Wissam A. Jassim

**Affiliations:** 1 Department of Computer and Communication Systems Engineering, Universiti Putra Malaysia, Serdang 43400, Selangor, Malaysia; 2 Department of Computer Engineering, University of Baghdad, Al-Jadriya 10071, Baghdad, Iraq; basheera412@yahoo.com or; 3 The Signal Processing Media Applications Group (SIGMEDIA), The global centre of excellence for digital content and media innovation (ADAPT Centre), School of Engineering, Trinity College Dublin, The University of Dublin, Dublin 2, Ireland; wissam.jassim@tcd.ie or

**Keywords:** shot boundary detection, temporal video segmentation, video content analysis, video browsing, video retrieval

## Abstract

The recent increase in the number of videos available in cyberspace is due to the availability of multimedia devices, highly developed communication technologies, and low-cost storage devices. These videos are simply stored in databases through text annotation. Content-based video browsing and retrieval are inefficient due to the method used to store videos in databases. Video databases are large in size and contain voluminous information, and these characteristics emphasize the need for automated video structure analyses. Shot boundary detection (SBD) is considered a substantial process of video browsing and retrieval. SBD aims to detect transition and their boundaries between consecutive shots; hence, shots with rich information are used in the content-based video indexing and retrieval. This paper presents a review of an extensive set for SBD approaches and their development. The advantages and disadvantages of each approach are comprehensively explored. The developed algorithms are discussed, and challenges and recommendations are presented.

## 1. Introduction

The rapid increase in the amount of multimedia data in cyberspace in the past two decades has prompted a swift increase in data transmission volume and repository size [1]. This increase in data has necessitated the exploration of effective techniques to process and store data content [2].

Video is the most consumed data type on the Internet. Videos consume a large amount of storage space, and they contain voluminous information [3]. Text, audio, and images are combined to constitute a video [4], so videos are large in size. The human brain gathers most information visually and can process visual media faster than it can process text. Thus, videos facilitate easy communication among individuals [5]. In the past two decades, computer performance, storage media availability, and the number of recording devices have increased considerably, resulting in the active uploading and viewing of videos at inconceivable rates [6]. For example, YouTube is the second most popular video sharing website (VSW). Statistics show that 300 hours of videos were uploaded every minute in 2016, and this figure presents a significant increase from the 72 hours of videos uploaded in 2015; furthermore, five billion hours of videos are being viewed daily. Video consumption increases at a rate of 300% annually. This growth is due to individuals and companies sharing their media through VSWs to broaden their audience. Moreover, individuals can now easily access the Internet as a result of the prevalence of mobile technology [2], which motivates them to upload videos to VSWs or social media. Readily available video editing software on computers and portable devices enable users to manipulate video contents by combining two or more videos, altering videos by adding other video contents, and omitting certain video parts. In addition, uploading videos to hosting sites is no longer restricted to skilled programmers, and this condition has resulted in video duplication. Video repetitions occur in many forms, such as downloading and re-uploading a video as it is, inserting logos, and covering copyrights by replacing video features (e.g., changing illumination or resizing video frames).

The unprecedented increase in the amount of video data has led to the improvement of relevant techniques for processing and storing large volumes of data [7] through the merging of multimedia data contents with their storage. For example, searching for an image from a data source using a text-based search engine is time consuming due to the usage of simple identifiers while ignoring the available information in the image itself [8]. Manual search is required to retrieve the appropriate image. To address this problem, a descriptor of the image content is utilized and merged with the data, similar to what occurs in Google’s image search. Notably, a video search engine remains unavailable, and this unavailability continues to motivate research on analogous video search engines based on video content [8].

Indexing and retrieval of multimedia information are performed to store, depict, and arrange multimedia data appropriately and swiftly [9]. Video structure analysis is fairly difficult owing to the following video attributes: (1) videos contain more information than images; (2) videos contain a large volume of raw data; and (3) videos lack or possess a very small prior structure. Multimedia databases, especially those for videos, created decades ago are comparatively smaller than current databases owing to the aforementioned characteristics, and annotation was performed manually based on keywords. Databases at present have ballooned in size and in the amount of video information they hold, thus establishing the need for automated video structure analysis without human involvement [3,9,10,11].

Video structure analysis involves content-based video indexing and retrieval (CBVIR) and aims to automate the management, indexing, and retrieval of videos [3]. CBVIR applications have expanded widely. These applications include browsing of video folders, news event analyses, digital museums, intelligent management of videos in VSWs, video surveillance [9], video error concealment [12], key frame extraction [13], and video event partitioning [14].

Shot boundary detection (SBD) also known as temporal video segmentation, is the first process in CBVIR, and its output significantly affects the subsequent processes. SBD performance influences the results and efficiency of the subsequent CBVIR modules, so SBD is considered a relevant stage in CBVIR [10,11,15,16,17,18,19]. The target of SBD is to partition a video into its basic units (shots) to be forwarded to the rest of the CBVIR modules for further analysis [20,21]. A shot is a continuous frames recorded by a single camera. A transition between two shots can be categorized into two types: hard (cut) and soft (gradual) transition.

SBD approaches can be divided broadly into two categories based on the feature extraction domain, namely compressed and uncompressed domain. For fast SBD algorithms, features are extracted from compressed domain because no decoding process for video frames are required. However, uncompressed domain have gained more attentions by researchers because of the vast amount of visual information in the video frames. Although SBD algorithms in the uncompressed domain are considered more reliable, more computational resources are required compared to compressed domain.

In general, the performance of a SBD algorithm is based on its ability to detect transitions (shot boundaries) in a video sequence. That is, SBD algorithm performance can be measured by its ability in detecting correct transition. Where, a SBD accuracy generally depends on the extracted features and their effectiveness of representing the the visual content of video frames [22]. The second factor that influences a SBD algorithm performance is the computational cost of the algorithm, that need to be reduced where in contrast, algorithm speed is increased. Note that, theoretically, within a shot, frames are very similar in terms of their visual content. Therefore, when shot transition is occurred, a change in similarity/dissimilarity values will be appeared. In hard transition (HT), a very high change in similarity/dissimilarity values, but for soft transition (ST) it is small [23]. Practically, there are some effects that appear in a video shot such as: flash lights or light variations, object/camera motion, camera operation (such as zooming, panning, and tilting), and similar background. These effects are highly provoking the accuracy of transitions detection and thus greatly impact on SBD algorithm performance. To fulfill the maximum efficiency, SBD should be able to detect shot transitions between two consecutive shots by, first, minimizing both false alarm signals (FASs), i.e., false positives, within a shot (intra-shot frames), and second, miss detects (MSD), i.e., false negatives, between two consecutive shots (inter-shot frames) during transition detection process. Currently, there is no complete solution to these problems or most of them in the same algorithm. That is, a favorable and efficient method for detecting transitions between shots remains unavailable despite the increasing attention devoted to SBD in the last two decades. This unavailability is due to the randomness and size of raw video data. Hence, a robust, efficient, automated SBD method is an urgent requirement [11,19].

Most of the existing reviews are not covering the recent advancements in the field of SBD. Therefore, it is necessary to have a novel and comprehensive review paper that presents and discusses the state-of-the-art algorithms in the field of SBD. This paper does not deal with high-level video analysis but on methods used to facilitate high-level tasks, which are SBD algorithms. Specifically, the mainly focusing of this paper on review and analyze different kinds of SBD algorithms that are implemented in the uncompressed domain following their accuracy rate, computational load, feature extraction technique, advantages, and disadvantages. Future research directions are also discussed. To provide a clear inspection of state-of-the-art SBD methods, their classifications and relations to one another are explored in detail according to previous work. In addition, recommendations related to the datasets and algorithms used in this work are provided for the benefit of researchers.

This paper is organized as follows: Section 2 introduces the fundamentals of SBD. Section 3 provides a comparison of compressed and uncompressed domains. Section 4 presents the SBD modules, and Section 5 presents a categorized survey on SBD approaches. Section 6 discusses the SBD evaluation metrics. Section 7 discusses the challenges in SBD and offers recommendations. Section 8 presents the SBD unrevlead issue and future direction. Section 9 presents the conclusion.

## 2. Fundamentals of SBD

### 2.1. Video Definition

**Figure 1:**
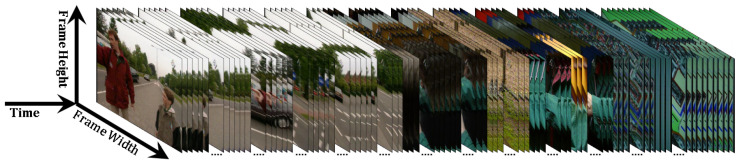
Video signal sample (Dataset: TRECVID 2007, Video ID: BG_2408, Frames’ indices: 151 to 613).

Text, audio, and image constitute the contents of a video data stream. Videos contain richer information compared with images, and their organization is not well defined [24]. This scenario highlights the need for video content analysis [25,26]. A video is a signal composed of a sequence of frames with a specific frame rate ($F_{rate}$ measured in frames per second or fps) accompanied by an audio track. A video is defined as a 3D signal in which the horizontal axis is the frame width ($N_{x}$) and the vertical axis is the frame height ($N_{y}$) representing the visual content. The third axis represents the variation in frame content along with time (*T* for total time or $N_{f}$ for number of frames $=T_{\mathrm{in}\;\mathrm{seconds}}\times F_{rate}$) [4,27], as shown in Figure 1. Hence, a point in a video is identified by its 2D positions (*x* and *y* pixel positions) and the time or frame index at which it occurs. A video can be described as

(1) $$V={\left\{f_{n}\right\}}_{n=1}^{N_{f}}$$

where $f_{n}$ is a video frame at index *n*. A video frame represents the visual perception of an object and/or locale at a specific time, that is, $n/F_{rate}$. Each pixel (*P*) in a video frame can be described as a function of frame index (*n*), location (*x* and *y*), and intensity (*r*) such that $P(f_{n},x,y,r)$ is the pixel intensity at locations *x* and *y* belonging to frame $f_{n}$. $1\leq x\leq N_{x}$, and $1\leq y\leq N_{y}$; $N_{x}$, and $N_{y}$ are the frame width, and frame height, respectively. Note that *r* is the number of bits used to represent each pixel in the frame (pixel bit depth).

### 2.2. Video Hierarchy

**Figure 2:**
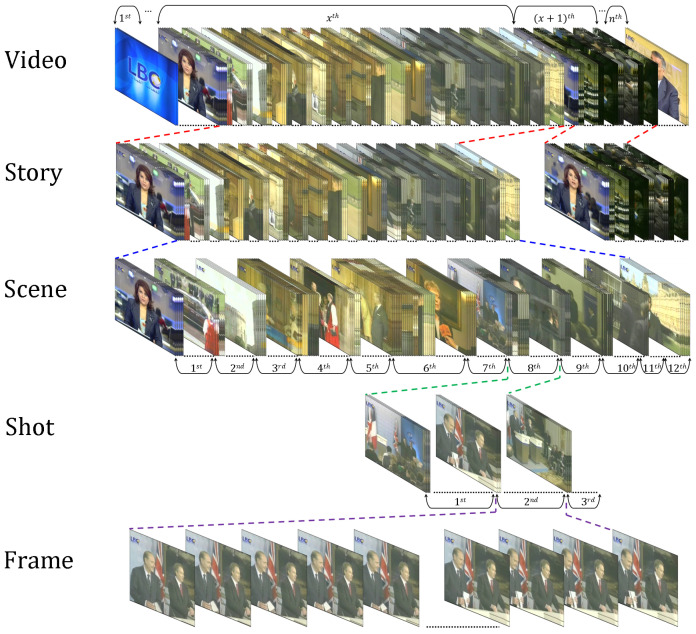
Video Hierarchy (Dataset: TRECVID 2005, Video ID: 20041119_140000_LBC_LBCNAHAR_ARB, Frames: 1, …, 104,895, Story frame ID: 1^st^ = 1, …, 1001, x^th^ = 36,293, …, 40,731, x + 1^th^ = 40,732, …, 41,589, …, n^th^ = 54,918, …, 104,895.

To some extent, video hierarchy is comparable to a book. A video consists of a single story (such as a football game) or multiple stories (such as news) [11]. A story is defined as a clip that captures a series of events or a continuous action, and it may be composed of several scenes. A scene is a pool of semantically related and temporally contiguous shots captured at multiple camera angles [16,28]. Figure 2 shows the hierarchy of a video. As previously mentioned, the hierarchy of a video closely resembles that of a document, such as a book consisting of chapters, which are similar to stories in a video [29]. Each chapter comprises sections similar to scenes. Sections consist of paragraphs similar to a video comprising shots. A paragraph is a group of interconnected sentences that are similar to the interconnected frames that constitute a shot in a video. Moreover, a sentence is composed of multiple words, similar to a shot being composed of frames. Each frame in a video represents a single image, while a shot represents a continuous sequence of frames captured by a single camera, as explained previously.

A shot is the building block of a video; it is a set of one or more frames grabbed continually (uninterruptedly) by a single recording device, and these frames symbolize an incessant action in time and space that shows a certain action or event [1,3,6,15,30,31]. A shot is also considered the smallest unit of temporal visual information [3,11,15]. The frames within a shot (intra-shot frames) contain similar information and visual features with temporal variations [32,33]. These variations in time between shot elements (i.e., frames) may cause small or large changes due to the action between start and stop marks [34]. These changes are due to the fact that a shot captures objects in the real world and the semantics, dynamics, and syntax of these objects are merged to obtain shot frames [3], such as object motion, camera motion, or camera operation. Moreover, a shot is supposed to comprise rigid objects or objects composed of rigid parts connected together [3]. Shots are classified into four types according to the object and/or camera motion; these types are static object with a static camera, static object with a dynamic camera, dynamic object with a static camera, and dynamic object with a dynamic camera [35]. A frame is the smallest unit that constitutes a shot. Hence, the shot and scene hierarchies are analogous to a sentence and paragraph. Shots are essential in depicting a story, and scenes are a necessary unit for a visual narrative [16]. Video frames are temporally ordered, but they are not independent [36].

### 2.3. Video Transition Types

The frontier between two shots is known as the boundary or transition. Concatenation between two or more shots is implemented in the video editing process (VEP) to create a video during the video production process (VPP) [37]. The digital video editing process allows for the creation of incalculable types of transition effects. Directors or individuals use VEP for stylistic effects. Generally, the frontiers between shots are of two types, namely, hard and soft transitions.

HT occurs when two successive shots are concatenated directly without any editing (special effects). This type of transition is also known as a cut or abrupt transition. HT is considered a sudden change from one shot to another. For example, in sitcom production, a sudden change between two persons conversing at the same locale is recorded by two cameras [3,11,38,39]. Moreover, HTs occur frequently between video shots [40]. Thus, we can deduce that HT occurs between the last frame of a shot and the first frame of the following shot.

**Figure 3:**
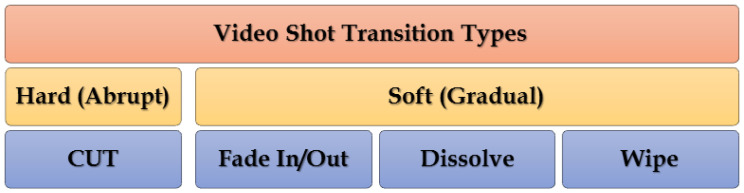
Video transition types.

By contrast, ST occurs when two shots are combined by utilizing special effects throughout the production course. ST may span two or more frames that are visually interdependent and contain truncated information [15,18,41]. Commonly, ST comprises several types of transitions, such as dissolve, wipe, and fade in/out [42] (see Figure 3).

As stated previously, VEP is used in VPP by individuals or institutes. VPP encompasses shooting and production processes. The former involves capturing shots, and the latter involves combining these shots to form a video [43]. During VEP, HT and ST are generated between shots to form a video.

A transition $T_{k}$ can be defined as the editing process between two successive shots $S_{k}=\langle f_{0@s_{k}},f_{1@s_{k}},\ldots ,f_{N_{\mathrm{sk}}-2@s_{k}},f_{N_{\mathrm{sk}}-1@s_{k}}\rangle$ and $S_{l}=\langle f_{0@l},f_{1@s_{l}},\ldots ,f_{N_{s}l-2@s_{l}},f_{N_{s}l-1@s_{l}}\rangle$. $N_{k}$ frames exist in transition $T_{k}$, such that $T_{k}=\langle f_{0@T_{k}},f_{1@T_{k}},\ldots ,f_{N_{k}-2@T_{k}},f_{N_{k}-1@T_{k}}\rangle$, in which the frames in the transition belong to the tail frames of $S_{k}$ and the head frames of $S_{l}$, where l=k+1.

**Figure 4:**
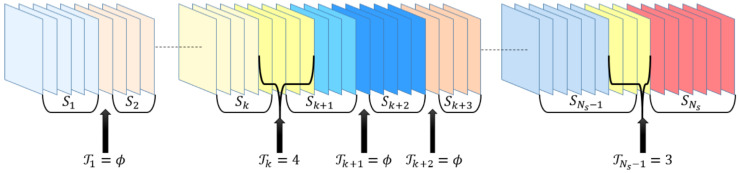
Transition period between frames.

For example, $T_{k}=\phi$ for HT, and $T_{k}\neq \phi$ for ST. These values may vary from 1 to $N_{k}$ frames, as illustrated in Figure 4. Detailed descriptions of HT and ST are provided in the following subsections.

#### 2.3.1. HT

**Figure 5:**
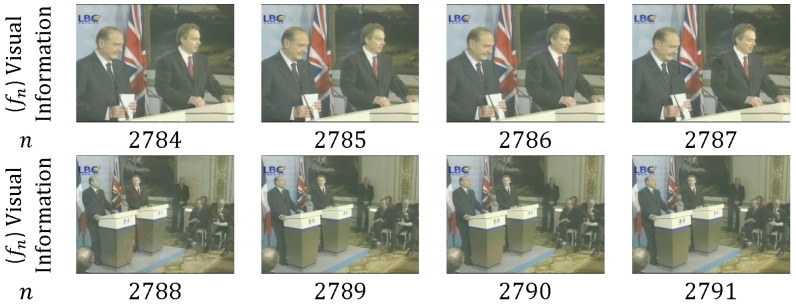
Example of HT. Frames are extracted from TRECVID 2007, Video ID: BG_2408.

HT is also known as a cut, a sharp transition, a sudden transition, and an abrupt change. HT refers to a sudden change in temporal visual information, in which two consecutive shots are attached without any VEP [44,45]. Figure 5 presents an example of HT, in which T=ϕ (no frames exist between two shots), which occurs between the last frame of the previous shot and the first frame of the next shot (between frames 2787 and 2788).

#### 2.3.2. ST

ST is also known as gradual or continuous transition. It is an artificial effect that may include one to tens of frames between two consecutive shots. ST is normally observed in television shows, movies, and advertisements. The frames in the transition period contain information from the two consecutive shots that are involved in this process; these two shots carry interrelated and inadequate visual information that are not utilized in video indexing and retrieval [11]. ST covers many types of transitions, including dissolve, fade in, fade out, and wipe.

In *dissolve*, the pixel intensity values gradually recede (diminish) from one shot $S_{k}$, and the pixel intensity gradually comes into view (appears) from the next shot $S_{k+1}$ (overlapping between shots that are partially visible) [46,47]. Thus, portions of both shots are shown at the same time by increasing and decreasing the pixel intensities of the frames for shots $S_{k+1}$ and $S_{k}$, respectively.

Dissolve transition can be described as [48]:

(2) $$T_{k}\left(m\right)=\alpha \left(m\right)S_{k}\left(m\right)+\beta \left(m\right)S_{k+1}\left(m\right)$$

where α(m) and β(m) are decreasing and increasing functions that usually satisfy α(m)+β(m)=1, m=1,…,M and M≥1, respectively.

**Figure 6:**
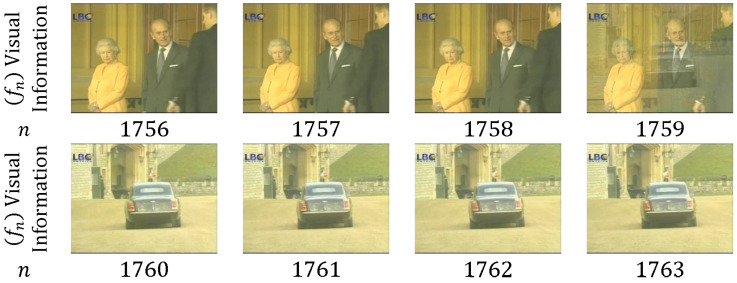
Dissolve transition example with one frame involved in transition (*n* = 1759). Frames are extracted from TRECVID 2007, Video ID: BG_2408.

**Figure 7:**
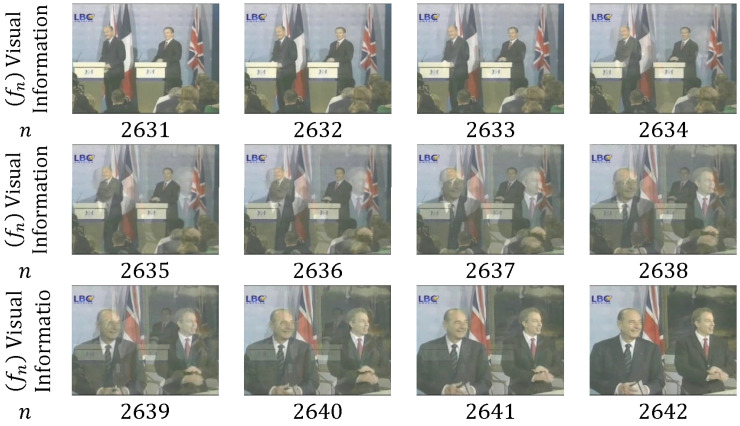
Dissolve transition example with 10 frames involved in transition (*n* = 2632–2641). Frames are extracted from TRECVID 2007, Video ID: BG_2408.

As shown in Figure 6, only one frame is in the dissolve transition between shots (*n* = 1759); α(m) and β(m) are approximately equal to 0.5 for each frame of the consecutive shots. Figure 7 depicts 10 frames that are utilized in the dissolve transition; α(m) decreases from 1 to 0, whereas β(m) increases from 0 to 1. Dissolve transitions may show nonlinearity in α(m) and β(m) [49].

**Figure 8:**
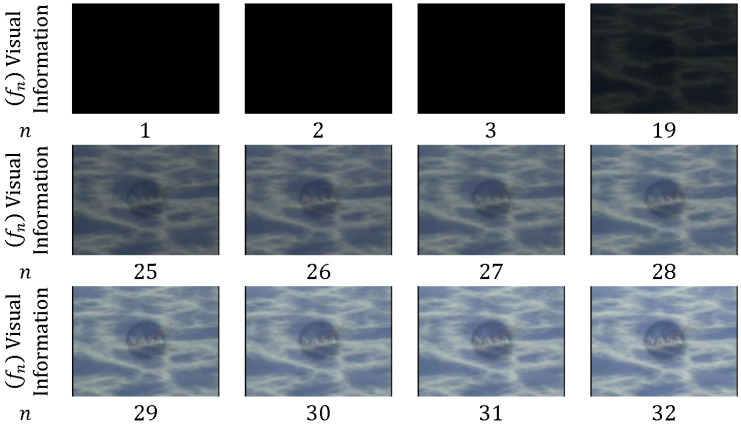
Fade-in transition example with 31 frames involved in transition (*n* = 1–32). Frames are extracted from TRECVID 2005, Video ID: 09-NASAConnect-AO.

In *fade in*, the pixel intensity values of shot $S_{k+1}$ gradually emerge from a fixed intensity frame. By contrast, previous shot $S_{k}$ is directly changed by the fixed intensity frame [50,51], as shown in Figure 8. Thus, only the frames at the end of shot $S_{k+1}$ are involved in fade-in transition, and no frames from shot $S_{k}$ are involved.

(3) $$T_{k}\left(m\right)=\gamma \left(m\right)f_{fixed}+\beta \left(m\right)S_{k+1}\left(m\right)$$

where γ(m) and β(m) are decreasing and increasing functions, respectively, that usually satisfy γ(m)+β(m)=1. $f_{fixed}$ is a fixed frame intensity, m=1,…,M, and M≥1.

**Figure 9:**
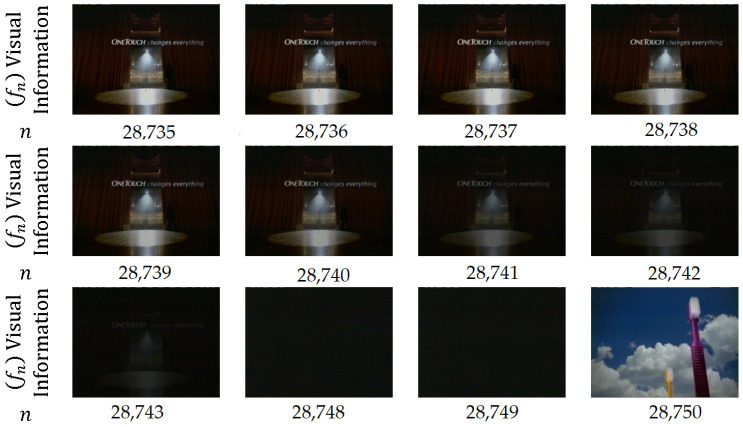
Fade-out transition example with 13 frames involved in transition (*n* = 28,735–28,750). Frames are extracted from TRECVID 2006, Video ID: 08-NBC_NIGHTLYNEWS_ENG.

In *fade out*, the pixel intensity values are gradually altered from one shot $S_{k}$ into a fixed intensity frame. The next shot $S_{k+1}$ instantaneously appears after the fixed intensity frame [52], as shown in Figure 9. Thus, only the frames at the end of shot $S_{k}$ are involved in fade-out transition, and no frames from shot $S_{k+1}$ are involved.

(4) $$T_{k}\left(m\right)=\alpha \left(m\right)S_{k}\left(m\right)+\gamma \left(m\right)f_{fixed}$$

where α(m) and γ(m) are decreasing and increasing functions, respectively, that usually satisfy α(m)+γ(m)=1; $f_{fixed}$ is a fixed frame intensity, m=1,…,M, and M≥1.

Briefly, fade in/out occurs when every pixel in the frame comes gradually into view from a single color or out of natural view into a single color.

(5) $$T_{k}\left(m\right)=\alpha \left(m\right)S_{k}\left(m\right)+\gamma \left(m\right)f_{fixed}+\beta \left(m\right)S_{k+1}\left(m\right)$$

where α(m) and γ(m) are decreasing and increasing functions, respectively, that satisfy α(m)+γ(m)=1 and β(m)=0 within the fade out transition period where m=1,…,M−L. In the case of fade in transition where m=M−L+1,…,M, β(m) and γ(m) represent the increasing and decreasing functions, respectively, that satisfy γ(m)+β(m)=1 and α(m)=0. Note that *L* is the frame number in the region where the fade out transition ends and the fade in transition starts.

**Figure 10:**
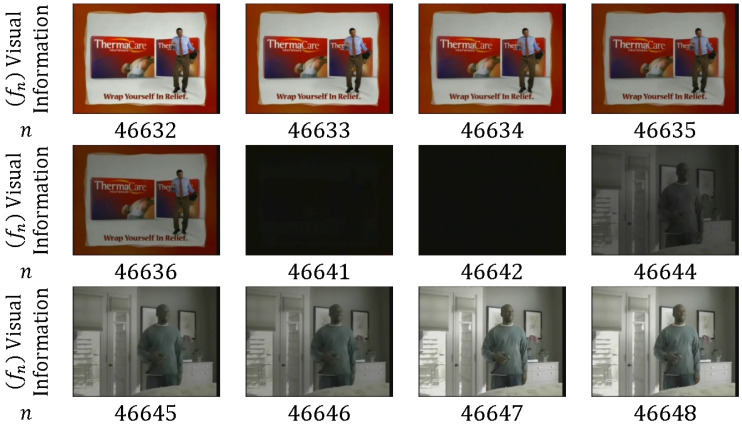
Fade out-in transition example with 15 frames involved in transition (*n* = 46,632–46,648). Frames are extracted from TRECVID 2005, Video ID: 02-20041106_110000_MSNBC_MSNBC.

In *fade out-in*, fade out starts from shot $S_{k}$ to the fixed frame, and then fade in starts thereafter from the fixed frame to shot $S_{k+1}$ [53], as shown in Figure 10. Thus, frames at the end of shot $S_{k}$ and starting frames from shot $S_{k+1}$ are involved in fade out–in transition.

**Figure 11:**
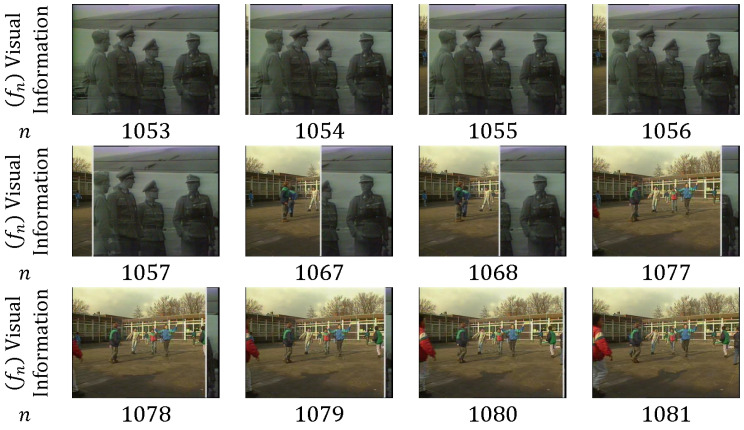
Wipe Transition example with 27 frames involved in transition (*n* = 1053–1081). Frames are extracted from TRECVID 2007, Video ID: BG_35187.

In *wipe*, the current shot pixels are progressively superseded by the corresponding pixels from the next shot by following an organized spatial pattern [54]. For example, gradually substituting column pixels from left to right of the frame is considered a typical wipe, as shown in Figure 11. Wipe transition can be described as follows:

(6) $$T_{k}\left(m\right)=\left\{\begin{array}{cc} S_{k}\left(x,y,m\right)\; & \forall (x,y)\in Uncovered\;wipe\;region\\ S_{k+1}\left(x,y,m\right)\; & \forall (x,y)\notin Uncovered\;wipe\;region \end{array}\right.$$

Other transition effects involve a combination of two or more types of the aforementioned transitions [55] which are infrequent and very challenging to detect [56].

ST differs from HT because of the high similarity between frames involved in the transition between two consecutive shots.

## 3. Compressed Doamin vs. Uncompressed Domain

Compressed domain (COD) and uncompressed domain (UCD) are the two main domains used in SBD. Several researchers have addressed the problem of SBD in COD, inasmuch as the obscurity of the decoding process leads to a fast processing algorithm. The utilized features, such as MPEG stream, are directly accessible from COD. These features include the coefficients of discrete cosine transform (DCT), macroblocks, and motion vectors. However, COD-based approaches are dependent on video compression standards and are not as accurate as methods that are based on UCD [34]. Moreover, video compression standards present low accuracy and reliability, particularly if videos are characterized by high motion, due to object/camera motion or camera operation [9,18,57,58]. Nowadays, several video compression standards, such as MPEG-1, MPEG-2, MPEG-4, H.261, and H.265, are available for use in such applications as video streaming and storage. If video quality is the major concern, I-frames might be too close to one another, or if a video is intended for streaming, I-frames might be too infrequent to save bandwidth. However, such sampling is prone to errors in cases of ST because a sample can easily be selected among the frames of an ST favorable for HT but not so for ST) [1,18,59]. These methods entail a low computation time because they work directly in COD; however, they cannot deal with visual data because they are highly dependent on the COD scheme [60]. Although performance is degraded in COD-based approaches, computational complexity can be reduced [61]. Owing to the demerits of COD, researchers are shifting their attention toward UCD because under this domain, the amount of visual information in the frame is vast and more valuable than that under COD [62].

## 4. SBD Modules

Generally, a SBD module encompasses three sub-modules: (1) feature of visual content; (2) construction of a continuity signal; and (3) classification of the continuity signal [4]. Each sub-module may include pre-processing and/or post-processing steps.

### 4.1. Representation of Visual Information (ROVI)

The representation of visual information (ROVI) for video frame $f_{n}$ is performed by extracting the visual features of video frames and acquiring a concise representation of the content for each frame [58], in which $Z_{n}=\Psi \left(f_{n}\right)$, where $Z_{n}$ is the extracted feature (feature domain) and Ψ is the function exercised for feature extraction. The aim of ROVI is to identify a suitable extraction method for features with two requirements: invariant and sensitive [4]. An invariant feature refers to the representation of frame visual information. The extracted feature is firm against the temporal variations of the frame, such as object and camera motion (OCM) [4]. By contrast, a sensitive feature can imitate the changes in frame content. In other words, invariant features remain stable within shots, whereas sensitive features present noticeable changes within shot transitions [4]. By combining invariant and sensitive features, a SBD with high accuracy in transition detection is achieved. In particular, many types of features are used in ROVI, and they include pixels, histograms, edges, motions, and statistics. Hence, the ROVI sub-module exerts a significant impact on SBD modules.

### 4.2. Construction of Dissimilarity/Similarity Signal (CDSS)

The CDSS sub-module is the intermediate stage between ROVI and classification sub-modules (transition detection) [58]. Usually, the distance (dissimilarity/similarity) between two successive frame features ($Z_{n}$ and $Z_{n+1}$) is computed (see Equation (7) for Minkowski distance). As a result, the stream of visual content is masqueraded into temporal signal(s) with one or multiple dimensions. In a perfect case, the dissimilarity signal carries high values at shot transitions and low values within the same shots. The opposite applies to the similarity signal. Owing to the randomness of video signals, vast amounts of disturbance exist in video signals, and these include object and/or camera motion and flash light occurrence, which affect the stability of dissimilarity/similarity signal. Addressing this issue entails embedding features of the current, previous, and next frames in CDSS.

(7) $$D\left(f_{n},f_{n+1}\right)={\left(\sum_{k=1}^{K}{\left|Z_{n}\left(k\right)-Z_{n+1}\left(k\right)\right|}^{p}\right)}^{1/p}$$

where *K* is the number of features and p>0. The Minkowski distance is also known as the $l_{p}-$ norm [63]. If p=1, then it is a city block distance, and if p=2, then it is Euclidean distance.

### 4.3. Classification of Dissimilarity/Similarity Signal (CLDS)

After the CDSS sub-module generates dissimilarity/similarity signal, CLDS is carried out to detect transitions and non-transitions between shots from dissimilarity/similarity signal. Classification based on a threshold is a simple direction [64]. In this strategy, the detected transition relies on fixed parameter(s). Methods based on a threshold are highly sensitive to many video genres because the threshold is selected based on one or more types of videos [64,65]. The limitations of approaches based on this scheme are shortage in differentiating between transitions and disturbance factors in dissimilarity/similarity signal with a fixed threshold(s). To overcome this drawback, SBD can be handled by assuming transition as a categorization problem. Machine learning-based approaches are utilized to eliminate the need for thresholds and embed multiple features. Machine learning schemes can be classified into two types: supervised and unsupervised. The issue of this method is the selection of an appropriate feature combination for SBD [66].

## 5. SBD approaches

In this section, the approaches used in SBD modules (i.e., ROVI, CDSS, and CLDS) are discussed collectively for each SBD algorithm. We introduce a survey on various SBD approaches that deal with HT and/or ST.

### 5.1. Pixel-Based Approach

The pixel-based approach (PBA) or pixel-wise comparison is used as a ROVI directly from the pixel intensities of video frames. PBA involves calculating the difference between two corresponding pixels (at location *x* and *y*) of two consecutive video frames ($f_{n}$ and $f_{n+1}$). In the next stage of PBA, the total sum of pixel differences is determined and compared with a threshold. A transition is declared if the sum exceeds the selected threshold [67].

The earliest researchers who implemented PBA for SBD are [68,69,70]. The researchers in [68] implemented PBA to locate HT by comparing the sum of the absolute differences of the total pixel (Equations (8) and (9)) with a threshold value. When the sum was greater than the threshold, HT was declared; otherwise, a frame’s shot was considered.

(8) $$D\left(f_{n},f_{n+1}\right)=\frac{1}{N_{x}N_{y}}\sum_{x=1}^{N_{x}}\sum_{y=1}^{N_{y}}\left|P\left(f_{n},x,y\right)-P\left(f_{n+1},x,y\right)\right|$$

(9) $$D\left(f_{n},f_{n+1}\right)=\frac{1}{N_{x}N_{y}}\sum_{x=1}^{N_{x}}\sum_{y=1}^{N_{y}}\sum_{k=1}^{C}\left|P\left(f_{n},x,y,c_{k}\right)-P\left(f_{n+1},x,y,c_{k}\right)\right|$$

where *C* is the number of color channels, P(·) is the pixel intensity, and $N_{x}$ and $N_{y}$ are the width and height of the frame, respectively. Equation (8) is used for single-intensity images (grayscale), and Equation (9) is used for multi-channel images.

The researchers in [69] modified the technique proposed in [68] to reduce the disturbance in dissimilarity signal. First, they compared the corresponding pixel differences of two successive frames to threshold $T_{1}$. When the partial difference exceeded $T_{1}$ (Equation (10)), they considered that pixel a change. Second, they summed up all the partial differences of the pixels and compared the result to a second threshold $T_{2}$ (Equation (11)) (the ratio of pixel change). When the value exceeded $T_{2}$, HT is detected.

(10) $$DP\left(f_{n},f_{n+1},x,y\right)=\left\{\begin{array}{cc} 1 & \mathrm{if}\;\left|P\left(f_{n},x,y\right)-P\left(f_{n+1},x,y\right)\right|>T_{1}\\ 0 & \mathrm{otherwise} \end{array}\right.$$

(11) $$D\left(f_{n},f_{n+1}\right)=\frac{1}{N_{x}N_{y}}\sum_{x=1}^{N_{x}}\sum_{y=1}^{N_{y}}DP(f_{n},f_{n+1},x,y)$$

Zhang et al. [70] found that a threshold should be selected manually for input videos to achieve good results. Manually adjusting a threshold is improbable in practice. Zhang et al. [70] followed the same technique used in [69] with a preprocessing step to detect HT and ST. They applied an averaging filter before conducting pixel comparison (Equations (10) and (11)). The averaging filter was used to replace the pixels in a frame with the average of its neighbor pixels. A 3×3 filter was used to average each video frame by convolving the filter with the entire frame. The reason for implementing this step was to reduce the noise and camera motion effects.

Another method was proposed in [24] to detect HT and ST by dividing the frame into 12 regions and determining the best match between regions in the current frame and the corresponding neighborhood regions in the next frame. The matching mechanism used mimics the mechanism utilized to extract motion vectors between two consecutive frames. The weighted sum of the sorted pixel differences for each region provides the frame difference measure [71]. STs are selected by generating a cumulative difference measure from consecutive values of the frame differences through the identification of sustained low-level increases in matched values.

Yeo and Liu [72] used DC images, which they considered low-resolution images of a video frame, extracted from video frames to detect HT and ST (fade only). They replaced the fixed threshold with a sliding window to compute the threshold (adaptive threshold).

PBA are highly sensitive to OCM, and they produce a high rate of false alarms (FAR). As a result of their dependency on spatial location, these techniques are particularly sensitive to motion, even global motion [73]. Although PBA techniques are highly sensitive to motion, missed detections (MSDs) occur [74]. For example, two adjacent frames within intra-shots with different pixel intensity disturbances can result in similar pixel differences. Furthermore, because of the high sensitivity of PBA techniques, intra-shots with camera motion can be incorrectly classified as gradual transitions. These methods rely on the threshold procedure, and they do not consider the temporal relation of dissimilarity/similarity signal. Table 1 presents a summary for the previously discussed PBA algorithms, their parameters settings and ability for detecting transitions.

### 5.2. Histogram-Based Approaches

Color histograms or histograms reflect the distribution of colors in an image. Histograms are considered substitutes for PBAs because they do not consider the spatial information of all color spaces. Hence, histograms, to some extent, are regarded as invariant to local motion or small global motion compared with PBAs [75,76].

Nagasaka and Tanaka [69] proposed a histogram-based approach (HBA) utilizing gray level for HT detection. However, the metric is not robust against temporary noise, flash light occurrence, and large object and/or camera motion. In [70], a technique called twin comparison was proposed. In this technique, the gray histograms of successive frames are computed and compared using a histogram difference metric (HDM) (Equation (12)) to detect HT and ST via low and high thresholds ($T_{L}$ and $T_{H}$, respectively). HT is detected when HDM is above $T_{H}$. By contrast, ST is detected when HDM is greater than $T_{L}$. In this condition, the computation of the accumulated difference continues until the difference falls below the low threshold $T_{L}$. The resulting accumulated difference is then compared with the high threshold $T_{H}$. The two thresholds (low and high) are automatically established according to the standard deviation and mean of the HDM for all video frames.

(12) $$HDM\left(f_{n},f_{n+1}\right)=\sum_{j=1}^{N_{H}}\left|H\left(f_{n},j\right)-H\left(f_{n+1},j\right)\right|$$

where $N_{H}$ is total number of possible gray levels and $H(f_{n},j)$ is the histogram value for the gray level *j* at frame *n*.

Another HBA was proposed in [77]. In this method, HDM is used to compute the histogram for each space in the RGB color space [78] after partitioning each frame into blocks on the basis of the following equation:

(13) $$HDM\left(f_{n},f_{n+1}\right)=\sum_{k=1}^{3}\sum_{b=1}^{N_{B}}\sum_{j=1}^{N_{H}}\frac{{\left(H\left(f_{n},j,c_{k},b\right)-H\left(f_{n+1},j,c_{k},b\right)\right)}^{2}}{H\left(f_{n},j,c_{k},b\right)-H\left(f_{n+1},j,c_{k},b\right)}$$

where $N_{B}$ is the total number of blocks and $H(f_{n},j,c_{k},b)$ is the histogram value for the $j^{\mathrm{th}}$ level in the channel *k* at the $b^{\mathrm{th}}$ block in the frame *n*.

In [79], two methods using a 64-bin grayscale histogram were presented. In the first method, a global histogram with an absolute difference is compared with a threshold. In the second method, video frames are partitioned into 16 regions. HDM is calculated for each block between successive frames. A transition is declared if the number of region differences that exceed the difference threshold is greater than the count threshold.

Lienhart [80] computed HDM based on a color histogram. A color histogram was implemented by discretizing the color component of RGB to $2^{U}$, resulting in components $\in [0,\;2^{U}-1]$. The discretization factor was used to reduce the sensitivity to low light and noise.

In [81], the HDMs of successive frames using histograms were computed on the basis of the hue component only. HDM can be described as follows:

(14) $$HDM\left(f_{n},f_{n+1}\right)=\frac{\sum_{j=1}^{N_{H}}\left|H\left(f_{n},j,c_{Hue}\right)-H\left(f_{n+1},j,c_{Hue}\right)\right|}{\sum_{j=1}^{N_{H}}H\left(f_{n+1},j,c_{Hue}\right)}\;$$

where $H(f_{n},j,c_{Hue})$ is the histogram value for the $j^{\mathrm{th}}$ level in the Hue channel at the frame *n*.

The authors also proposed a modification to the previous method by utilizing a block-based approach and using only six bits for the RGB histogram. This modification was realized by extracting the two most significant bits from each color space. HDM was computed on the basis of the blocks instead of the global histograms. The HDM acquired for each block was compared with a threshold to detect shot changes as follows:

(15) $$HDM\left(f_{n},f_{n+1}\right)=\sum_{b=1}^{N_{B}}\frac{\sum_{j=1}^{N_{H}}\left|H\left(f_{n},j\right)-H\left(f_{n+1},j\right)\right|}{\sum_{j=1}^{N_{H}}H\left(f_{n+1},j\right)}$$

where $N_{H}=64$, $N_{B}$ is the total number of blocks and $H(f_{n},j)$ is the histogram value for the $j^{\mathrm{th}}$ level in the quantized RGB space at frame *n*.

Ahmed and Karmouch [82] improved the previous algorithm by considering adaptive temporal skip as an alternative to fixed temporal skip. They compared frames $f_{n}$ and $f_{n+m}$. When HDM was greater than a threshold, they used $m=\frac{n+m}{2}$ in the new temporal skip. They repeated the process until m=n+1, which they considered a shot transition between $f_{n}$ and $f_{m}$.

(16) $$HDM\left(f_{n},f_{n+m}\right)=\sum_{b=1}^{N_{B}}\frac{\sum_{j=1}^{N_{H}}\left|H\left(f_{n},j\right)-H\left(f_{n+m},j\right)\right|}{\sum_{j=1}^{N_{H}}H\left(f_{n+m},j\right)}$$

where *m* is the temporal skip.

Gargi et al. [31] presented an HDM with various color spaces for histogram and distance measures. These color spaces included RGB, HSV, YIQ, L*a*b, L*u*v*, and Munsell [83,84]. For the distance metric, they implemented bin to bin, chi-squared ($\chi^{2}$), and histogram intersection. Their results showed that YIQ, L*a*b*, and Munsell outperformed HSV and L*u*v*, whereas the RGB scores were the lowest. The highest recall and precision for HT were 79% and 88%, respectively, whereas those for ST were 31% and 8%, respectively. The experiment was performed on a dataset consisted of 959 HTs and 262 STs. The video sequences of the dataset were complex with graphical effects and high dynamic motion.

Thounaojam et al. [85] proposed a shot detection approach based on genetic algorithm (GA) and fuzzy logic. The membership functions of the fuzzy system were computed with the use of GA by considering pre-observed values for shot boundaries. RGB color spaces with a normalized histogram between two consecutive frames were computed as follows:

(17) $$HDM_{i}=1-\left(\frac{1}{3N}\right)\left[\sum_{c\in R,G,B}\sum_{j=1}^{256}min\left(H\left(f_{i},j,c\right),\left(f_{i+1},j,c\right)\right)\right]$$

where $H(f_{n},j,c)$ is the histogram value for the $j^{\mathrm{th}}$ level in the channel *c* at the frame *n*, and *N* is the number of pixels.

Five videos from TRECVID 2001 were used to evaluate the proposed system. The overall average recall and precision for HT and ST were 93.1% and 88.7%, respectively. Notably, the ST types in TRECVID 2001 are mostly dissolve transitions, and only a few fade transitions are included.

Mas and Fernandez [86] used only four MSBs from R, G, and B spaces to obtain 4096 levels of histogram for detecting HT, dissolve, and fade. They also found no significant difference between RGB (red, green, blue) and HSV (hue, saturation, value) color spaces, which led to a noticeable improvement. City block distance between two color histograms was measured to obtain HDM. To detect HT, the HDM was convolved with a rectangular signal (size 13), and the result was compared with a threshold. For dissolve and fade transitions, the convolved HDM was utilized to locate the local maxima. Mathematical morphology operators were applied to the convolved signal to obtain the start and end of ST. Then, within the opening signal, they looked for the number of succeeding values that exceeded the structuring element duration. They explained that the method based on color histogram is still sensitive to camera motion, such as panning or zooming. Hence, refinement was implemented to remove false positives caused by camera motion or large moving objects in a scene.

Qian et al. [87] proposed a fade detector based on the accumulative histogram difference (see Equation (18)). The proposed method was implemented using gray frames in UCD and DC images in COD with a 64-bin histogram. Six cases of fade transition and their properties were discussed. The results showed that UCD has better recall and precision than COD.

(18) $$AHD_{n}\left(j\right)=\sum_{l=0}^{j}\Delta H_{n}\left(l\right)$$

where $\Delta H_{n}\left(x\right)=H_{n+1}\left(x\right)-H_{n}\left(x\right)$, and AHD is the accumulative histogram difference.

Ji et al. [88] presented a dissolve detection method based on accumulative histogram difference and support points for transition with a temporal window. All the frames in the observation window perform multiple operations on the binary image to ensure that the pixels have the characteristics of monotone properties.

In [37], fuzzy logic was used to generate a color histogram for HT and ST (fade and dissolve) detection. Transition detection was performed after video transformation to eliminate negative effects on SBD. The L*a*b* color space with 26 fuzzy rules was utilized to generate a 15-bin fuzzy color histogram. Two thresholds were utilized to detect HT and ST transitions. The algorithm was evaluated using the TRECVID 2008 dataset. The overall recall and precision of the algorithm reached 71.65% and 83.48%, respectively.

In [30], a SBD method based on a color histogram computed from a just-noticeable difference (JND) was proposed. The concept of JND refers to the process of mapping the RGB color space into a new color space with three orthogonal axes $J_{R}$, $J_{G}$, and $J_{B}$ which describe the JND on the respective R, G, and B axes. The values of the new color space are varied for each axis, which are in the range (0,24) for red, (0,26) for blue, and (0,28) for green [89]. The similarity between two successive frames from a JND color model was computed using histogram intersection. A sliding window-based adaptive threshold was used to detect HT and ST (dissolve and fade).

In [90], a different method for detecting transitions using a third-order polynomial curve and a color histogram was presented. In this method, each frame is decoded and resized to 240×180. Then, the RGB color space is converted into HSV color space and gray level. Subsequently, a 256-bin histogram is extracted from each space (H, S, V, and gray). The computed histograms are then sorted in ascending order and fitted using a third-order polynomial. The city block distance is determined between the feature vectors of successive features to form a dissimilarity signal. The feature vector is formed from four parts: (1) first non-zero value in the sorted histogram; (2) first non-zero forward difference in the sorted histogram; (3) polynomial value at x=1, where *x* is the polynomial variable; and (4) highest value in the histogram curve. The feature vector is formed from the four portions according to the weighted sum. The detection part is based on a preset threshold value such that a transition is identified if the dissimilarity signal is higher than a predefined threshold. The drawback of this algorithm is the implantation of a histogram that is sensitive to flashlights, similar backgrounds, and dark frames.

In [91], a three-stage approach based on the multilevel difference of color histograms (MDCH) was proposed. First, candidate HT and ST were detected using two self-adaptive thresholds based on a sliding window with a size of 15. ST transformed to HT, so ST can be managed in the same manner as HT. Second, the local maximum difference of the MDCH generated by shot boundaries was detected to eliminate the disturbances caused by object motion, flash, and camera zoom. Third, a voting mechanism was utilized in the final detection. HSV color space and Euclidean distance were employed in the algorithm with a five-level MDCH. Four videos from TRECVID 2001 were used for evaluation.

An HBA was used by Park et al. [92] to study the effect of an adaptive threshold on SBD. In the study, 45 bins from hue space and 64 bins from saturation were considered. The video frame was resized to 240×135 pixels. The adaptive threshold was computed using the similarity of the adjacent frame and a fixed threshold value. Recall and precision improved by 6.3% and 2.0%, respectively, with a maximum recall and precision of 82.3% and 85.5%, respectively. The researchers also studied the effect of resizing video frames on SBD by using a fixed threshold value [93]. Video frame resizing slightly affected recall and precision.

In [94], SBD was implemented as the first stage in keyframe extraction. RGB was quantized to eight intensities and eight bins for each channel. CDSS was computed using city block and compared with a fixed threshold to detect HT only.

HBAs are not as sensitive to object and/or camera motion as PBAs due to the obscurity of the spatial distribution of video frames. However, large object and/or camera motion cause a change in signal construction, similar to that in ST. In such a case, the detection of a false positive is declared as ST [20,41]. In addition, histograms are sensitive to flash light occurrence (illuminance changes), which also leads to false positives. A histogram difference remains sensitive to camera motion, such as panning, tilting, or zooming [40].

Distinguishing the shots within the same scene is insufficient [4]. In other words, two consecutive frames belonging to different shot frames (long scene) may exhibit the same color distribution, leading to a false negative (misdetection). Distinguishing between dissolve transition and motion is also problematic [95].

HBAs are established based on the assumption that two consecutive frames within a shot comprising steady objects and backgrounds present minimal diversity in their histograms. Unlike PBAs, HBAs are not overly sensitive to motion because they do not take the changes in the spatial distribution of frames into consideration. However, the assumption in establishing HBAs emphasizes the drawback of these approaches. In HBAs, two frames belong to different neighboring shots. The histograms of these frames are comparable, whereas their contents are completely or partially different. This characteristic leads to a measure similar to that of object and/or camera motion. Consequently, using HBAs to detect all HTs without incurring false positives and false negatives (misdetection) is a serious challenge. Despite their weaknesses, HBAs are widely used because of the tolerable trade-off between accuracy and computation cost.

For picture-in-picture transitions, change in small region (CSR), the histograms of two consecutive frames are expected to show similarities because of the minimal change in the frames. Table 2 summarizes the HBA algorithms in literature, their parameter settings and transition detection ability.

### 5.3. Edge-Based Approaches

Edge-based approaches (EBAs) consider a low-level feature of a frame. These approaches are implemented to detect HT and ST. In EBAs, transition is declared when the locations of the edges of the current frame exhibit a large difference with the edges of the previous frame that have disappeared. Edge detection (including new and previous edges), edge change ratio (ECR), and motion compensation are the required processes for computing edge changes. Although EBAs demonstrate the viability of edges (frame feature), their performance is unsatisfactory compared with that of simple HBAs [96,97]. Moreover, EBAs are expensive, and they do not outperform HBAs. Nevertheless, these approaches can remove false positives resulting from flash light occurrence (sudden illumination changes) because they are more invariant to various illumination changes than HBAs. Given this property, the authors in [98,99] proposed a flash light occurrence detector based on edge features and used this detector to filter out candidate transitions.

The first work that used EBA for HT and ST was [100]. The authors smoothened the image with a Gaussian smoothing filter with radius *r* and then determined the gradient value using a Canny operator [101]. Afterward, the image was dilated. The previous steps are denoted as $E\left(\cdot \right)$ and $\bar{E}\left(\cdot \right)$, where E(·) is the edge detection and $\bar{E}\left(\cdot \right)$ is the dilated version of E(·). $E(f_{n})$ and $E(f_{n+1})$ are the edges for frames $f_{n}$ and $f_{n+1}$, respectively. The fraction of the edge pixels in $E(f_{n+1})$ is subsequently determined. This fraction is greater than fixed distance *r* from the closest edge pixel in $E(f_{n})$, and it is labeled as $\rho_{\mathrm{in}}$ (measures the ratio of entering edge pixels). $\rho_{\mathrm{in}}$ should be a large value during cut and fade-in transitions or at the end of a dissolve transition. $\rho_{\mathrm{out}}$ is the ratio of edge pixels in $E_{f_{n}}$, that is, the distance greater than *r* away from the closest edge pixel in $E_{f_{n+1}}$. $\rho_{\mathrm{out}}$ measures the proportion of exiting edge pixels. It should be large during fade out and cut transitions or at the start of a dissolve transition. Similarity measure ρ is described as follows:

(19) $$\rho =max\left(\rho_{in},\rho_{out}\right)$$

(20) $$\rho_{in}=1-\frac{\sum_{x=0}^{N_{x}-1}\sum_{y=0}^{N_{y}-1}\bar{E}\left(f_{n}\left(x+\Delta x,y+\Delta y\right)\right)E\left(f_{n+1}\left(x,y\right)\right)}{\sum_{x=0}^{N_{x}-1}\sum_{y=0}^{N_{y}-1}E\left(f_{n}\left(x+\Delta x,y+\Delta y\right)\right)}$$

(21) $$\rho_{out}=1-\frac{\sum_{x=0}^{N_{x}-1}\sum_{y=0}^{N_{y}-1}E\left(f_{n}\left(x+\Delta x,y+\Delta y\right)\right)\bar{E}\left(f_{n+1}\left(x,y\right)\right)}{\sum_{x=0}^{N_{x}-1}\sum_{y=0}^{N_{y}-1}E\left(f_{n}\left(x,y\right)\right)}$$

Throughout the dissolve transition, the edges of objects gradually vanish, whereas new object edges become gradually apparent. Moreover, the edges appear gradually in fade-in transition and disappear gradually in fade-out transition. This characteristic is revealed by using ECR to detect HTs; it was later extended to detect STs [102].

During the first part of the dissolve transition, $\rho_{\mathrm{out}}$ dominates over $\rho_{\mathrm{in}}$, whereas during the second part, $\rho_{\mathrm{in}}$ is larger than $\rho_{\mathrm{out}}$. For fade-in transition, $\rho_{\mathrm{in}}$ is the most predominant, and, for fade-out transition, $\rho_{\mathrm{out}}$ is the most predominant. The result is an increment in ρ (ECR) for the ST period, which is utilized to detect STs. Although EBAs can achieve good detection of STs, the FAS rates are unsatisfactorily high [80,103].

EBAs are utilized for transitions only. Thus, false detection occurs during camera operation, including zooming. In addition, multiple object movements produce false positives. If shots show extreme movement during HT, a false dissolve transition occurs. In the transition from a static scene to extreme camera movement, a cut may be misclassified as a fade transition.

In [104], a method utilizing an EBA based on wavelet transform was proposed. First, the authors spatially subsampled frames via 2D wavelet transform to extract edges for temporally subsampled frames to construct a continuity signal. Second, this signal was parsed after applying a 1D wavelet transform. A hard change in the signal was considered a candidate transition. Third, the candidate segment was further analyzed to detect transition by applying 1D wavelet transform. EBA was applied for the temporal subsampled frames in a block-based manner. The computed edge points for each block were used between two successive frames. Candidate frames were declared as a transition when a hard change in the continuity signal occurred. When the edge points had large values, the difference between inter-frames was applied using 1D wavelet transform.

An EBA was used in [80] to detect a dissolve transition. The authors performed the detection by utilizing a Canny edge detector, and they distinguished between strong and weak edges by using two threshold values. After the refined edges were obtained, a dissolve transition was declared when the local minimum occurred for the current edge value. The global motion between frames was estimated and then applied to match the frames before perceiving the entering and exiting edge pixels. However, this algorithm cannot overcome the presence of multiple fast-moving objects. According to the authors, an additional drawback of this approach is the number of false positives resulting from the limitations of the EBA. In particular, rapid illuminance changes in the overall inter-frame brightness and extremely dark or bright frames may lead to FAS. This method was improved and implemented later for dissolve detection in [105] by utilizing edge-based contrast instead of ECR to capture and emphasize the loss in contrast and/or sharpness.

Heng and Ngan, in their original [106] and extended work [107], proposed a method based on an EBA. They presented the concept of an object’s edge by considering the pixels close to the edge. A matching of the edges of an object between two consecutive frames was performed. Then, a transition was declared by utilizing the ratio of the object’s edge that was permanent over time and the total number of edges.

An EBA based on a Robert edge detector [108] for detecting fade-in and fade-out transitions was proposed in [109]. First, the authors identified the frame edges by comparing gradients with a fixed threshold. Second, they determined the total number of edges that appeared. When a frame without edges occurred, fade in or fade out was declared. The interval bounded by two HTs was regarded as the region considered for such transitions.

In sum, EBAs are considered less reliable than other methods, such as a simple histogram, in terms of computational cost and performance. With regard to the computational cost, EBAs involve edge detection and pre-processing, such as motion compensation. Despite the improvement in transition detection, EBAs are still prone to high rates of false alarms resulting from many factors, such as zoom camera operations. A summary of the discussed EBA algorithms, their parameters setting, and ability for detecting transition is provided in Table 3.

### 5.4. Transform-Based Approaches

Transform-based approaches (TBAs) involve transforming a signal (frame) from the time (spatial) domain into the transform domain. Discrete transform is a useful tool in communication and signal processing [110,111,112]. It allows the viewing of signals in different domains and provides a massive shift in terms of its powerful capability to analyze the components of various signals. Discrete transforms are characterized by their energy compaction capability and other properties [113]. Discrete Fourier transform (DFT) and discrete cosine transform (DCT) are examples of discrete transforms. The difference between transforms is determined by the type of the transform (polynomial) basis function. Basis functions are used to extract significant features of signals [114]. For example, DFT uses a set of complex and natural harmonically related exponential functions, whereas DCT is based on a cosine function with real values from −1 to 1.

Porter et al. [115] proposed a method for HT detection by determining the correlation between two consecutive frames. First, each frame was partitioned into blocks with a size of 32×32. Second, the correlation in the frequency domain (see Equation (22)) between each block in frame $f_{n}$ with the corresponding block and neighbor blocks in the next frame $f_{n+1}$ was considered, along with the largest coefficient value of the normalized correlation. Third, the correlation was identified in the frequency domain. The transition was detected when a local minimum was present.

(22) $$\rho \left(a\right)=\frac{F^{-1}\left\{{\hat{x}}_{1}\left(w\right)\;{\hat{x}}_{2}^{*}\left(w\right)\;\right\}}{\sqrt{\int {\left|{\hat{x}}_{1}\left(w\right)\right|}^{2}dw\cdot \int {\left|{\hat{x}}_{2}\left(w\right)\right|}^{2}dw}}$$

where *a* is the spatial coordinate, *w* is the spatial frequency coordinate, ${\hat{x}}_{i}\left(w\right)$ is the Fourier transform of $x_{i}\left(a\right)$, * denotes the complex conjugate, and $F^{-1}$ is the inverse Fourier transform operator. To compute a CDSS, the mean and standard deviation are computed for all correlation peaks.

In [116], Vlachos computed the phase correlation for overlapped blocks between successive frames. The video frames were converted to CIF format, and only the luminance component was utilized. HT was declared when a low correlation occurred between two consecutive frames of the CDSS.

In [117], which is an extension of [115], the maximum normalized correlation coefficient was sought after applying a high pass filter to each image before correlation. The similarity median ($M_{n}$) of the obtained normalized correlation was calculated, and the average of the previous measure from the last detected HT was computed as $\bar{M}=\sum_{i=1}^{n-1}M_{i}/\left(n-1\right)$. HT was declared when $\bar{M}-M_{n}>threshold$. For fade-out transition (respectively, in), if $M_{n}$ dropped (respectively, lifted) to 0 and the standard deviation of the frame pixel values decreased (respectively, increased) before the current value, then the first frame of the fade-out transition (respectively, in) was marked at the point where the standard deviation began to decrease (respectively, increased). The end of the fade-out transition (respectively, in) was identified by correlating the video frame with an image with constant pixel values, resulting in $M_{n}=0\;\left(\mathrm{respectively},\;1\right)$. A dissolve transition was detected by extending the procedure of detecting fade in (out) via the median vaxlue $M_{n}$ and the median of the block variances. The reported results showed that the recall and precision for HT and ST were 91% and 90% and 88% and 77%, respectively. The experiment was performed on a dataset consisted of 10 collected videos with 450 HTs and 267 STs, and the ground truth is hand-labeled for comparison.

In [118], Cooper et al. computed the self-similarity between the features of each frame and those of the entire video frame to form a similarity matrix *S* using a cosine nonlinear similarity measure.

(23) $$S\left(i,j\right)=\frac{\langle \vec{v_{i}},\vec{v_{j}}\rangle }{∥\vec{v_{i}}∥\;∥\vec{v_{j}}∥}$$

where $\vec{v_{n}}$ is the feature vector of frame $f_{n}$. The feature vector was formed by concatenating the low-order coefficients of each frame color channel after the transformation to the DCT domain. A conversion from RGB color space to Ohta color space, the method in [119] was implemented prior to the transformation. Similarity matrix *S* was correlated with a Gaussian checkboard kernel along the main diagonal to form a 1D function. The resulting signal was compared with the thresholds defined previously to detect HT and ST.

Urhan et al. [120] later modified the phase correlation algorithm of [116] by using spatially sub-sampled video frames to detect HT for archive films. The effect of noise, flashlight, camera rotation and zooming, and local motion were considered in the phase correlation. Adaptive local and global thresholds were used to detect HT. In [121], Priya and Domnic proposed a SBD method for HT detection in a video sequence using OP. Walsh–Hadamard OP basis functions were used to extract edge strength frames. Each frame was resized to 256×256 (the Walsh–Hadamard OP coefficient order should be $2^{m}$) and then divided into blocks of size 4×4. Only two basis functions from OP were used to compute the edge strengths by transforming the basis functions from a matrix form into a vector form. The value for each block was computed by applying the dot product with each block intensity as follows:

(24) $$\begin{array}{c} Z_{m}=\left\{z_{1}^{m}=\langle K_{m},V_{1}\rangle ,z_{2}^{m}=\langle K_{m},V_{2}\rangle \right\}\\ m=1,2,\ldots ,number\;of\;blocks \end{array}$$

where $K_{m}$ is the $m^{\mathrm{th}}$ block and $V_{j}$ is the orthogonal vector. CDSS was computed by the sum of absolute differences between the features of the blocks. Then, CDSS was applied to a transition detection procedure based on a threshold to detect HT.

The main drawback of the method proposed in [121] is that utilizing a threshold in detection could not be generalized for all videos. Moreover, the author mentioned the existence of FASs between gradual transition and object/camera motion along with MSDs for some gradual transition. Additionally, missed detection is possible for low-intensity frames (dark frames) because of the lack of frame intensity normalization. Table 4 provides a summary of the discussed TBA algorithms, parameter setting, transform used, and transition type detection ability.

### 5.5. Motion-Based Approaches

Motion-based approaches involve computing motion vectors by block matching consecutive frame blocks (block matching algorithm or BMA) to differentiate between transitions and camera operations, such as zoom or pan. Motion vectors can be extracted from compressed video sequences (i.e., MPEG). However, BMA performed as a part of MPEG encoding for vector selection is based on the compression efficiency, which in turn leads to regularly unsuitable vectors. The selection of unsuitable motion vectors causes a decline in SBD accuracy [79].

In [24], BMA was used to match a block in the current frame with all other blocks in the next frame. The results were combined to differentiate between a transition and considerable camera motion within a shot because shots exhibiting camera motion may be considered ST. The detection of camera zoom and pan operations increases the accuracy of the SBD algorithm. Boreczky and Rowe [79] improved the previous method of Shahraray [24] by dividing frames into 4×3 blocks and using block matching with a search window of 24×18 to extract motion vectors. HT was declared when the match value obtained from the motion vectors was greater than a threshold. Bounthemy et al. [122] estimated the dominant motion in an image represented by a 2D affine model and impended in a SBD module.

In [105], Lienhart implemented the motion estimator proposed in [123] to differentiate (detect FASs) between transitions and camera operations (pan and zoom). Bruno and Pellerin [124] proposed a linear motion prediction method based on wavelet coefficients, which were computed directly from two successive frames.

In [125], Priya and Domnic extended their previous work based on OP [121] by implementing a motion strength vector computed between compensated and original frames using the BMA presented in [126]. The achieved motion strength feature was fused with edge, texture, and color features through a feature weighting method. Two methods were presented to detect HT and ST: procedure transition detection and statistical machine learning (SML).

Motion-based algorithms are unsuitable in the uncompressed domain because estimating motion vectors requires significant computational power [127]. In a typical scenario, BMA matches blocks with a specified neighbor region. For an accurate motion estimation, each block should be matched with all blocks of the next frame, which lead to a large and unreasonable computational cost.

### 5.6. Statistical-Based Approaches

Statistical-based approaches (SBAs) are considered an extension of the aforementioned approaches, such that the statistical properties are computed for global or local frame features. Mean, median, and standard deviation are examples of statistical properties. These properties are used to model the activity within shots and between shots. SBAs are tolerant of noise to some extent, but they are considered slow because of the statistical computation complexity. In addition, SBAs generate many false positives [79].

Jain et al. [128] proposed a method by computing the mean and standard deviation of intensity images for regions.

SBA was proposed in [129] by utilizing the variance of intensities for all video frames to detect dissolve transition by perceiving two peaks from the second-order difference instead of detecting parabolic shapes, which is considered a problem for continuity signals formed by the variance of frames.

The authors in [130] reported that the real dissolve transition with large spikes is not always obvious. They assumed that a monastically increasing pattern should be observed in the first derivative with a dissolve transition. They also detected fade-in and fade-out transitions on the basis of the same method, in which the frames within a fade show small to zero variance during fade-out transition and vice versa during fade-in transition [51].

Hanjalic [40] proposed a statistical model for HT and dissolve detection. First, the discontinuity values were computed by using motion compensation features and metrics. DC images from MPEG-1 standard were used in the model. Owing to the small size of the DC images, a block of size 4×4 and a maximum displacement of four pixels in the motion compensation algorithm were adopted. Second, the behavior of the intensity variance and temporal boundary patterns were added to the detector as additional information to reduce the effect of luminance variation on detection performance. Finally, a threshold technique was used to detect transitions. No shot duration of less than 22 frames was assumed in this work.

Miadowicz [131] implemented color moments for story tracking. Mean, standard deviation, and skew were the color moments considered for HT, dissolve, and fade detection.

Despite the good performance of the aforementioned methods, not all motion curves can be accurately fitted by a B-spline polynomial. Thus, utilizing goodness of fit for ST detection is not reliable. The assumptions for an ideal transition, such as linear transition model and transition without motion, cannot be generalized for real videos [132].

The discussed TBA algorithms are summarized in Table 5. In addition, Table 5 provides the parameters settings for each algorithm and their transition detection ability.

### 5.7. Different Aspects of SBD Approaches

Owing to the importance of SBD, many researchers have presented algorithms to boost the accuracy of SBD for HT and ST.

#### 5.7.1. Video Rhythm Based Algorithms

In [133], Chung et al. proposed a technique for HT and ST (specifically wipe and dissolve transitions) known as video rhythm by transforming 3D video data (*V*) into a 2D image ($V_{R}$) such that the pixels along the horizontal or vertical planes are uniformly sampled along a reference line in the corresponding direction of the video frames. They proposed that HT appears as a vertical line in $V_{R}$ and as a continuous curve in wipe transition. Video data *V* of $N_{f}$ frames have a frame size of ($N_{x},N_{y})$. The intensity level of pixel (x,y) is denoted by $P(f_{k},x,y)$, and $V_{R}$ is a grayscale image of size (z,t) such that $V_{R}\left(z,t\right)=T\left[f_{t},t\right]$, where $T\left[\cdot \right]$ can be any transform. The intensity level of pixel (x,y) is obtained by transforming frame $f_{k}$ (i.e., $P(f_{k},x,y)$) and *t*. For example, diagonal frame data are implemented via $V_{R}\left(z,t\right)={\left.\;\;P(f_{t},x,y)\right|}_{x=1,\ldots ,N_{x},\;y=x}$. Three algorithms are implemented to detect HT, wipe, and dissolve transitions.

Ngo et al. [134] modified the video rhythm method. In their work, horizontal, vertical, and diagonal temporal slices were extracted from each frame with four channel components (red, green, blue, and luminance). Each slice component formed a 2D image (one for space and one for time). From these slices, any change was considered to denote the existence of a transition. To detect HT, the authors sought local minima using a temporal filtering technique adopted from Ferman and Teklap [135]. For wipe transition, the energy levels of three spatio-temporal slices were computed, and the candidate wipe transition was located. The color histograms of two neighbor blocks of the candidate region were subsequently compared. Hough transform [136] was performed to locate the wipe transition boundaries if the histogram difference exceeded the threshold. In dissolve transition, the authors implemented the statistical characteristics of intensity from the slices and applied the algorithm presented in [137]. The latter transition could not be easily detected because the rhythm was intertwined between two successive shots as a result of the change in coherence being difficult to distinguish according to color–texture properties.

The algorithm suffers from mislaying (misdetection) CSR detection in addition to its sensitivity to camera and object motions. In other words, the algorithm exhibits high sensitivity and low invariance. Additionally, if a video sequence comprises a constant frame box in one or all sides of an entire video or a part of it, MSDs occur. In other cases, subtitles or transcripts lead to FASs.

#### 5.7.2. Linear Algebra Based Algorithms

In [27,58], Amiri and Fathy presented SBD based on linear algebra to achieve satisfactory performance. In [27], QR-decomposition properties were utilized to identify the probability function that matches video transitions.

In [58], Gaussian functions of ST, in addition to a distance function for eigenvalues, were modeled by using the linear algebra properties of generalized eigenvalue decomposition (GED). To find HT and ST, the authors performed a time domain analysis of these functions. Regardless of the reported performance in a small dataset, these techniques are considered to have high complexity as a result of the implementation of an extensive matrix operation [138]. This resulted from the injection of GED by a 500-feature vector, which was achieved by partitioning each video frame into four equally sized blocks and extracting a 3D histogram of RGB color space from each block. Afterward, GED was applied to the extracting feature matrix. These algorithms generated FASs when flash light occurred due to the considered sampling rate of frames (5 fps). MSD could be expected because of the similar backgrounds during HT or ST. Additionally, these methods are time consuming owing to the high number of matrix operations they entail [138].

#### 5.7.3. Information Based Algorithms

A SBD method was proposed by Černeková et al. [50] on the basis of information theory; this method is applied prior to keyframe extraction. Joint entropy and mutual information were separately computed for each RGB component between successive frames. A predefined threshold was utilized to detect HT and fade transition. However, the limitation of this method is its sensitivity to commercials which produces FASs and HTs between two shots of similar color distributions are missed.

Baber et al. [39] utilized speeded-up robust features (SURF) and entropy (local features, and global features) to find HT and ST (fade only, as explicitly mentioned). Video frames were resized to 128×128 as a preprocessing step. After resizing the frames, the entropy of each frame was computed using an intensity histogram. Then, the difference of the entropies between two consecutive frames was compared with a threshold. From this step, candidate shots were selected. These candidate shots were further processed by utilizing SURF keypoints and computing the matching score between two frames, which was compared with another threshold to polish the candidate frames. Fade transition was based on the computed entropy. Run length encoding was executed on a binary sequence extracted from the entropy as follows:

(25) $$\begin{array}{c} r_{0}=0;\;\\ r_{i}=\left\{\begin{array}{cc} 1 & e_{i}>e_{i+1}\;\\ 0 & e_{i}<e_{i+1}\\ r_{i-1} & e_{i}=e_{i+1} \end{array}\right. \end{array}$$

Thereafter, run length encoding was developed by extracting a vector of three features (F1, F2, and F3). Two thresholds were utilized with the extracted feature vector to detect fade-in and fade-out transitions. The limitation of this method is that it can only be applied to fade transition. SURF generates an error in keypoint matching in cases of high-speed camera motion. SURF also leads to many matching errors due to frame resizing. In addition, the assumption of fade transition interval is inappropriate because, in practice, transitions intervals is unknown.

#### 5.7.4. Deep Learning Based Algorithms

Recently, employing deep learning algorithms in the field of computer vision received much attention from academics and industries. Convolutional Neural Networks (CNN) is one of the most important deep learning algorithms due to its significant abilities to extract high level features from images and video frames [139]. The architectures of the CNN algorithms are suitable to be implemented by GPUs that are able to handle matrix and vector operations in parallel.

A SBD algorithm based on CNN is presented in [33]. An adaptive threshold process was employed as a preprocessing stage to select candidate segments with a group size of five frames. A similar design of the preprocessing stage is illustrated in [15]. Thereafter, the candidate segments were fed to a CNN with seven layers to detect the HTs and STs. The CNN was trained using the ImageNet dataset on interpretable TAGs of 1000 classes. The five classes corresponding to the five highest output probabilities were considered as high level features or TAGs for each frame. The detection process is based on the assumption that the TAGs are similar within a shot and dissimilar between two shots. To perform the HT detection between frames *n* and n+1 for segments with a length of six frames, the following constraint was proposed to validate the HT detection:

(26) $$|\left(T\left(f_{n-3}\right)\cap T\left(f_{n-1}\right)\cap T\left(f_{n+2}\right)\cap T\left(f_{n+4}\right)\right)|\leq 1$$

where $T\left(f_{n}\right)$ is the TAGs of the $n^{\mathrm{th}}$ frame. In terms of the STs detection for segments with a length of 11 frames, each candidate segment was first divided into two portions TF(t) and TB(t), which represent the combined TAGs of the start and end portions at the $t^{\mathrm{th}}$ candidate segment. TF(t) and TB(t) are defined as follows:

(27) $$\begin{array}{c} TF(t)=T(s-5)\cap T(s-3)\cap T(s-1) \end{array}$$

(28) $$\begin{array}{c} TB(t)=T(e+5)\cap T(e+3)\cap T(e+1) \end{array}$$

where *s* and *e* are the start and end frame index of the $t^{\mathrm{th}}$ segment. To detect the ST at the $t^{\mathrm{th}}$ segment, the following condition should be considered:

(29) TF(t)∩TB(t)=ϕ

Recently, a feature extraction approach based on CNN was proposed in [140]. A preprocessing step similar to that of [15,33] was employed to group the possible candidate segments. A standard CNN was trained using the ImageNet dataset with 80000 iterations. The feature set was extracted from the output of the 6^th^ layer. Then, the Cosine similarity measure (ψ) was employed to construct the similarity signal between feature sets of two consecutive frames. The detection of HTs at the $n^{\mathrm{th}}$ frame is based on three conditions.In the case of ST, absolute difference was employed between the first and last frame of a segment. The obtained similarity measure is shown to be as an inverted isosceles triangle for an ideal ST. Thus, the problem of detecting ST is moved to pattern matching as in [15].

The above mentioned CNN-based SBD algorithms achieved a noticeable accuracy. Note that the performances were evaluated using seven videos taken from the TRECVID 2001 dataset.

#### 5.7.5. Frame Skipping Technique Based Algorithms

Li et al. [38] proposed a framework based on the absolute differences of pixels. Frame skipping (fragmenting a video into portions) was proposed to reduce the computational cost of SBD in selecting candidate transitions when the difference exceeds the local threshold given by:

(30) $$T_{L}=1.1\;\mu_{L}+0.6\;\left(\frac{\mu_{G}}{\mu_{L}}\right)\;\sigma_{L}$$

where $T_{L}$ is the local threshold, $\mu_{L}$ is the local mean, $\sigma_{L}$ is the local standard deviation, and $\mu_{G}$ is the global mean for all the distances. A refinement process was implemented to determine whether the selected segments were actual transitions.

In this approach, the locations of cut and gradual transitions are determined approximately [15]. Moreover, the frame skipping interval to reduce the number of processed frames in a limited scope and the selection of a fixed interval of 20 frames are unreasonable [18]. This method is unsuitable for real-time applications because it requires buffering 21 frames [12]. In addition, it is sensitive to flash, color changes, and object/camera motion because of the application of PBA using luminance to compare two frames [18]. In experiments, this method shows a high MSD for HT and ST, thus disproving the assumption that “FASs are much better than MSD” in thresholding. This characteristic is explained as follows: all candidate portions go through additional processes to eliminate FASs, whereas MSD portions that contain transitions are discarded as non-boundary portions.

Lu and Shi [15] proposed a fast SBD scheme. In the first process, a PBA and a modified local threshold for skipping (Equation (31)) were used, as presented by Li et al. [38], with frame skipping of 21. They added constraints to collect several candidate segments to overcome the problem of MSD in [38]. After collecting candidates, a filter out process was used to eliminate FASs from the first stage. This scheme still yields FASs despite the MSD.

(31) $$T_{L}=\mu_{L}+0.7\;\left(1+ln\left(\frac{\mu_{G}}{\mu_{L}}\right)\right)\;\sigma_{L}$$

A SBD algorithm based on SURF was proposed by Birinci et al. [141] to detect HT and ST. SURF [142] was used for feature keypoint extraction and matching from each frame with a frame skipping ($N_{skip}$) of 1 s (which varied according to the frame rate of the video, i.e., 25 frames when $F_{rate}=$25 fps) to accelerate the process of SURF resulting from its high computational cost. Thereafter, the extracted keypoints were processed via structural analysis to verify the frame (dis)similarity. The number of matched features was used to compute the fraction of matched keypoints to the total number of keypoints. This process was performed to avoid bias resulting from the variation in the extracted keypoints from each frame. The authors followed a top–down approach, that is, overview followed by zoom and filter. The algorithm uses frame skipping to reduce the computation cost; however, for a simple camera motion, a candidate transition requires further zooming to filter out the candidate (unnecessary computational load). FASs occur due to blurred frames caused by camera motion. Another drawback is the emergence of MSD due to low-intensity (dark) frames.

Bhaumik et al. [143] proposed a method to detect dissolve transitions by utilizing two stages. In the first stage, the candidate transitions were distinguished by recognizing the parabolic patterns generated by the fuzzy entropy of the frames. To detect false positives in the candidate transitions, the authors designed four sub-stages based on thresholds in the second stage. The four sub-stages were edge detector, fuzzy hostility index, statistics of pixel values, and ECR.

In [144], a SBD algorithm based on GA was presented. The proposed algorithm makes use of the ECR proposed by Zabih et al. [100]. A preprocessing step was performed to sample a video at 2 fps based on the assumption that the shot lengths were longer than 15 frames. The proposed algorithm showed many FASs due to camera operations as well as MSDs due to frame skipping (sub-sampling) and the assumption that the shots were longer than 15 frames. For example, in TRECVID 2006, video ID 12 shows 14 shots with less than 15 frames and 71 transitions; the accuracy of the algorithm in this case is reduced.

A two-round SBD method was presented by Jiang et al. [41]. In the first round, a window with a size of 19 was used, and a method similar to frame skipping was applied by finding the similarities among the first, middle, and last frames in the defined window. The candidate was identified by computing the histogram and pixel differences of the frames using two thresholds. Each frame was unevenly divided into nine blocks that were then arranged into three groups. Later, a second round was initiated using Scale-invariant feature transform (SIFT) to eliminate false alarms. Multiple weights were applied for each group before computing the overall dissimilarity for the pixels and histograms (the YUV color space was used to compute the histogram). For a gradual transition, multiple thresholds were used to find the gradual transitions from the dissimilarity signals of the histograms and pixels.

#### 5.7.6. Mixed Method Approaches

In [145], a PBA was used by resizing the video frame aside from morphological information to detect HT and ST (i.e., dissolve and fade transitions). The pre-processing step (frame resizing) was implemented by reducing the size of the frame from 352×240 to 44×30. The authors also utilized the HSV color space by converting the color space from RGB to HSV. The V space was solely used for luminance processing. The authors counted the pixel difference ($C_{P}\left(f_{n}\right)$), which equated to over 128 (considered the threshold). If the counted value $C_{P}\left(f_{n}\right)$ exceeded the previous $C_{P}\left(f_{n-1}\right)$ value, then a cut was declared. Gradual detection began when the variation in the pixels increased above a specific threshold and ended when the variation became lower than the threshold. To refine the results, the authors applied a dilation filter with a size of 7×7. A candidate was validated if the number of its pixels, which changed after the dilation, exceeded the threshold. The threshold was considered to be half of the entire frame. Although image resolution decreased to speed up the computation and guard against excessive camera and object motions, MSD arose due to the similar backgrounds of the resized frames. Additionally, high FASs tended to occur because of high object motion.

An algorithm based on multiple features was presented by Lian [71]. These features are pixel, histogram, and motion. The motion features were calculated based on BMA. To detect HT, the author defined the YUV color space for pixel difference, block histogram difference, and block matching based on the absolute histogram difference along with four thresholds. In the absence of HT between nonconsecutive frames, the second stage of ST detection was executed. In this case, a new distance was measured between nonconsecutive frames as a replacement for consecutive frames. If each measure exceeded the specified threshold (four thresholds) for gradual transition, then it was declared as ST and then passed to a flash light detector according to the histogram to detect flash light occurrence. Through this method based on many threshold values, specifying the threshold for each video group yields good results. Furthermore, a non-negligible amount of MSD was observed in transitions for changes in small regions (CSRs) and fast dissolve transitions. FASs also occurred due to frame blur occurrence.

A unified SBD model for HT and ST detection, excluding wipe transition because of model linearity, was suggested by Mohanta et al. [146]. The model is based on the estimated transition parameter for the current frame using the previous and next frames. The model makes use of global and local features. A color histogram was used for global features, whereas edge and motion matrices were implemented for local features with a block size of 5×5 within a search area of 15×15. A feature vector with a size of 108 was constructed from the global and local features and then fed to a neural network to classify the input as follows: no transition, HT, fade in/out, and dissolve. Finally, a post-processing step was utilized to reduce the FASs and MSDs from the classifier (as reported) resulting from the misclassification between motions and transitions. An assumption that shot length should not be less than 30 frames was adopted in the post-processing stage. The drawbacks of this algorithm are as follows. First, the linear transition model is inappropriate for cases involving fast OCM or operations. Second, the algorithm cannot detect wipe transition. Third, the computational cost of the algorithm is extremely high due to edge detection and matching, followed by block matching with a small size and large search area, and parameter estimation for each feature (global and local). Fourth, the assumption of shot length cannot be less than 30 frames is insufficient. For instance, video 12 in TRECVID 2005 comprises 36 shots, which are composed of less than 30 frames.

A method based on visual bag of words for SBD was proposed by Lankinen and Kämäräinen [147]. This method mainly relies on local descriptors. SIFT was utilized to calculate the local descriptors. A codebook was generated from two million features extracted from ImageNet [148]. The video was sub-sampled at 1 fps. Then, the keypoints were extracted to form a descriptor. Thereafter, the nearest match in the codebook was found for each frame descriptor utilizing a KD tree search. Histograms were computed from the codes and the city block distance then applied between current and previous frame histograms. The difference between the computed distances was determined and compared with a threshold to detect transitions. The limitation of this work is its massive computational cost due to the use of the SIFT algorithm and codebook search. Moreover, this algorithm is not able to achieve an acceptable accuracy, especially for STs [125].

A radon transform-based SBD method was proposed by Bhalotra and Patil [149]. An image intensity was projected along the radial line at a particular angle. The projections of the frames differed in a video sequence, and they were used to identify the continuity signal from the radon projections of the frames. The continuity signal was compared with a threshold to find the transition between frames. This method involves a high computational cost owing to the radon transform for each frame. Moreover, it fails to differentiate between object/camera motions.

FFT was utilized by Miene et al. [150] for grayscale frames along with two metrics from the spatial domain to detect transitions.

An iterative algorithm was introduced by Grana and Cucchiara [62]. The algorithm was designed for transitions with linear behavior to deal with HT and ST. The goal was to find an optimum transition length and extremities by utilizing the fewest number of parameters [60], unlike the method presented by Bescos that utilizes 20 parameters for transition detection [62]. The proposed iterative method implements PBA and HBA and exhibits distinctive capabilities in comparison with common methods [8,27]. In this method, the transition center is identified by considering different frame steps. The proposed method iteratively measures the linearity behavior of a transition by minimizing the error function [151]. Although an adaptive threshold is implemented in the preprocessing stage, the results are not always satisfactory [15]. Moreover, the computational cost is high [38]. Owing to the algorithm assumption that features within shots are constant, the algorithm suffers from camera/object motions [151,152].

Ling et al. [44] employed multiple features, namely, edge histograms, histograms in HSV space, and pixel intensity difference. These features were used to form an input feature vector in SVM, which was used to detect HT and ST. As temporal features are not considered, the algorithms are sensitive to flash light and object motions in real-world applications.

A supervised classification-based method for SBD was suggested by Cooper et al. [61]. In this method, new intermediate features are identified by using pairwise inter-frame similarity, a global histogram of 32 bins, and a local histogram of eight bins. These features are fed to a supervised classifier based on the binary k-nearest neighbor algorithm to detect transitions.

An algorithm for detecting gradual transitions was proposed by Yoo et al. [153] on the basis of edge information. The edges of each video frame were computed using a Sobel operator [154], and the average edge gradients were calculated. Then, the variances were computed from nine non-overlapping frames. Dissolve and fade transitions were detected using the variance distribution of edge information. Two thresholds were used to detect ST.

Iwan and Thom [21] proposed a method that used audio and video separately for content analysis to detect the end-of-act in a circus for Circus OZ videos. In the first stage, the start and end of applause were detected based on audio content analysis via the extraction of eight audio features (MFCC, compactness, fraction of low energy frames, beat, spectral, frequency, zero crossings, and root mean square) for every 3 sec. Weka data mining was used in [155] to build a classification model and a multilayer perception classifier for two classes (clap and non-clap). In the second stage, the start and end of the black color were detected based on two conditions: (1) the black frame duration is greater than a preset temporal threshold; and (2) the ratio of black color is greater than a predefined threshold. To differentiate between the clapping sounds occurring at the middle or end of the act, this stage was proposed. In this stage, HBA and a clustering algorithm were used to measure the similarity between two adjacent frames. Multiple frames formed on each side of the detected applause sound. Hue and saturation channels were used to generate a color histogram of 30 and 32 bins, respectively. The reported average recall and precision were 92.27% and 49.05%, respectively. This method achieves low precision, and it is designed for special circus performances.

Dutta et al. [23] presented two algorithms for SBD on the basis of the variation pattern of similarity signal based on local edge detection. The algorithms are based on the assumption that a “shot transition model assumes that frames in a shot are visually very similar with respect to a reference frame, and the discontinuity in similarity values occurs at a shot transition,” i.e., linear slope. The first algorithm is a straightforward implementation of the transition model, and the transitions are declared based on the threshold value. Linear slope approximation was used as an alternative to the absolute value of the similarity values. A post-processing technique was used to reduce false detection according to the histogram computed between the first frame (respectively, last) and the estimated frame of the first (respectively, last) frame based on the last frame (respectively, first). Frame estimation was performed by matching a 16×16 block within a search window of 33×33. This algorithm was designed to find HT, fade, and dissolve. In the evaluation part, a relaxation of ±5 was considered for HT and ST.

Finally, Table 6 demonstrates a comparison among different SBD algorithms based on features employed, frame skipping, dataset used, accuracy (precision, recall and F1 score measures), and computational cost. Note that the reported results presented in Table 6 are listed as given in the corresponding work. From the table, it can be observed that the algorithms used frame skipping technique have low computational cost with an acceptable accuracy as in [15]. Although some algorithms utilize frame skipping, they show a moderate computational cost because of the computation complexity of the features used such as SIFT, SURF, and Harris. Obviously, algorithms that show a high computational cost such as [22,125] gain a remarkable accuracy compared to other algorithms. For [125], the computational load is due to utilizing local features and motion compensation, and, for [22], it is due to the number of decomposition levels and local features that are used for each color space.

## 6. SBD Evaluation Metrics

Two prospective metrics, namely, accuracy and computational cost, are used to evaluate the performance of SBD algorithms. In most cases, accuracy and computational cost cannot be achieved with a single method or algorithm. This distinction between the two metrics is due to the fact that the improvement of one metric is achieved on the account of the second one. For example, improving accuracy requires additional computations in the designed method. This case is common when designing an offline algorithm. On the contrary, real-time applications entail low computational costs on the account of accuracy [157].

### 6.1. SBD Accuracy Metrics

Assume that test video V has a number of HTs ($N_{HT}$) and STs ($N_{ST}$). Let G(·) be a SBD algorithm to be evaluated, and let the output of algorithm *G* be an array A that holds the start locations of the detected transitions, the end locations of the detected transitions, and the transition types. As a result, the array has a size of 3, as determined by $N_{TD}$, where $N_{TD}$ is the number of detected transitions for video V using algorithm A, i.e., A=G(V). Each test video comprises ground truth array $A_{Ground}$ with a size of 3 as determined by ($N_{HT}+N_{ST}$). The ground truth is either associated with the videos in the standard dataset or built by researchers for the test video. The ground truth and detection arrays are occasionally omitted by researchers if the types of STs do not need to be distinguished.

The number of detected transitions, $N_{TD}$, is equal to the number of correctly detected transitions and the number of falsely detected transitions, i.e., $N_{Correct}$ and $N_{FAS}$, respectively. The difference between the correctly detected transitions and the number of transitions in the ground truth is the number of missed transitions, $N_{MSD}$.

Precision and recall are the two measures used to evaluate SBD algorithms. Precision P is the number of correctly detected transitions (reported as true positives) relative to the number of detected transitions (reported as true positives and false positives).

(32) $$\begin{array}{ccc} P & = & \frac{N_{Corrrect}}{N_{Correct}+N_{FAS}} \end{array}$$

(33) $$\begin{array}{ccc} P & = & \frac{N_{Corrrect}}{N_{TD}} \end{array}$$

Recall R is defined as the number of correctly detected transitions (reported as true positives) relative to the number of transitions in the ground truth (reported as true positives and true negatives).

(34) $$\begin{array}{ccc} R & = & \frac{N_{Correct}}{N_{Correct}+N_{MSD}} \end{array}$$

(35) $$\begin{array}{ccc} R & = & \frac{N_{Correct}}{N_{Ground}} \end{array}$$

where $N_{\mathrm{Ground}}$ is the total number of transitions in the ground truth of the video.

Another measure called the F-score (harmonic mean) [158,159] that combines precision and recall to achieve one score is written as

$$F_{\beta }=\frac{(1+\beta^{2})\;P\;R}{\beta^{2}\;P+R}\;,$$

where β is a non-negative value. The most widely used value of β is 1, which is called the F1-score.

(36) $$F_{\beta }=\frac{(1+\beta^{2})\;P\;R}{\beta^{2}\;P+R}$$

β is a non-negative value. The most used value of β is 1 which is called F1-score:

(37) $$\begin{array}{ccc} F1 & = & \frac{2\;P\;R}{P+R} \end{array}$$

(38) $$\begin{array}{ccc} F1 & = & \frac{2\;N_{Correct}}{N_{TD}+N_{Ground}} \end{array}$$

Detecting all correct transitions without FASs and MSD is considered an ideal case. Several algorithms are designed with multiple stages [18,39,41], where the first stage aims to collect candidates that include all correct transitions with FASs. A low precision and a high recall are achieved from the first stage. Thereafter, a second stage or post-processing stage is utilized to filter out FASs and thereby improve precision and maintain recall. In other words, the refinement stage should ideally remove false alarms without affecting correct transitions. For example, a test video has $N_{Ground}=100$ transitions, and a SBD algorithm involves two stages (S1 and S2). In the first stage, $N_{TD}=200$ with $N_{Correct}=90$, and $N_{FAS}=110$ and $N_{MSD}=10$. The second stage filters out the candidate transitions ideally ($N_{FAS}=0$). If the refinement stage is not ideal, then not all FASs are removed, and several correct transitions are removed, e.g., $N_{FAS}=10$ and $N_{Correct}=85$ then $N_{MSD}=15$. In other words, precision increases while recall retains its value in an ideal case. In a non-ideal case, precision increases, and recall slightly decreases in comparison with the results achieved from the first stage.

### 6.2. SBD Computation Cost

Computational cost is rarely discussed by researchers when evaluating algorithm designs. However, computational cost is an important attribute of SBD algorithms, especially in real-time applications. An extensive survey concerning computational cost was presented in [160]. The computational cost of a SBD algorithm was measured in terms of the number of operations required per frame. These operations included mathematical and logical operations that considered absolute values.

### 6.3. Dataset

The datasets used to evaluate SBD algorithms are crucial and require careful examination and selection. Standard datasets are appropriate in evaluation because the datasets of researchers are limited by factors such as availability, representativeness, and ground truth availability. Moreover, the datasets created by researchers cannot always be accessed. The inaccessibility of these datasets is sometimes due to domain or website issues. Not all datasets are representative and include all types of transitions to be evaluated, thus leading to biased results. For example, a SBD algorithm may be designed to handle all types of transitions, but the dataset only includes two types of transitions. Ground truths are not associated with researchers’ datasets, and thus, building these datasets is difficult and time consuming for other researchers. TRECVID evaluation [161] is co-sponsored by the National Institute of Standards and Technology. TRECVID was established to evaluate and benchmark SBD tasks [161], and it has contributed to the improvement of SBD algorithms [27]. The most famous datasets of TRECVID for SBD are TRECVID 2001, TRECVID 2005, TRECVID 2006, and TRECVID 2007. These datasets are freely provided by the institute after a designated application form is submitted. In particular, the TRECVID 2001 dataset includes HT, dissolve, and fade transitions. TRECVID 2007 is mainly designed for HT and only has 6% STs [39]. TRECVID 2005 and 2006 datasets are considered the most challenging datasets owing to the diversity of their transitions.

The requirements for a representative and trustworthy comparison of SBD algorithms are as follows: similar conditions, same datasets, and standard datasets. These requirements must be met to achieve a fair comparison, reduce the time and effort needed to rebuild algorithm(s), and achieve accuracy against multiple interpretations and implementations [157]. Moreover, videos used for training should be mentioned explicitly or be excluded from comparisons; that is, only testing videos should be used to ensure a fair and representative comparison.

## 7. Open Challenges

Different aspects of SBD algorithms were discussed and analyzed in this work. The review indicates that the presented algorithms generate important findings and offer good contributions to this field. These different approaches improve the performance of SBD algorithms; however, an accurate and efficient SBD algorithm is still needed to handle the issues in the results of previous studies. Challenges must also be addressed, as discussed in the following subsections.

### 7.1. Sudden Illuminance Change

FASs or false positives are common in the results of previous studies. FASs are declared as transitions due to the disturbances in the CDSS, the technique used in CLDS, the ROVI implemented, and sudden illuminance changes.

Sudden illuminance changes occur due to flash lights or lighting effects. Several studies have been conducted to tackle the flash light issue, and their focus was on flash lights [87,98,162] as a post-processing stage in a SBD algorithm [72,163] or the features assumed to be invariant to light effects [58]. Although these methods can tackle sudden illuminance changes, they still suffer from this issue. For example, Amiri and Fathy [58] stated that false alarms caused by illuminance changes are not completely detected. In another example, the method presented by Chen et al. [162] can only deal with flash changes when the duration is short and when the contents of a video are similar before and after the flash occurrence. The FASs resulting from illuminance changes can be solved by: (1) identifying the location of the light source; (2) considering temporal relations about the suspected transition; (3) selecting features that are invariant to lighting effects; (4) deliberating the color space prior to feature extraction; and (5) differentiating between flash lights and fade transitions with a white or high-intensity frame.

### 7.2. Dim Lighting Frames

Dim lighting frames (DLFs) are frames with low brightness. To the best of our knowledge, no previous study has discussed the effect of the transition between shots with DLFs, especially for videos with multiple lighting effect and DLFs. The same is not true in the case of videos with approximately constant lighting throughout shots. The transition between such shots would be mislaid (MSD) due to the high similarity in the CDSS between the frames of the current and next shots. Correctly detecting the transitions between shots with DLFs entails the following: (1) a pre-processing step is required to detect the lightness level of each frame; and (2) if DLF frames are detected, then either a procedure to increase the lightness or a sub-process to process these frames is needed.

### 7.3. Comparable Background Frames

Comparable background frames are likely to emerge between shots. Transitions bounded by shots with comparable background frames show high similarity, which in turn lead to MSDs if an improper ROVI, such as HBA and global PBA, is utilized.

### 7.4. Object and Camera Motion

Various OCMs render SBD algorithms incapable of distinguishing between smooth OCM and STs; this issue remains open because of the lack of a clear modeling of spatial features [58]. For example, a smooth OCM is counted as ST, which increases FAR, or ST is regarded as OCM, which increases the number of MSD. A fast OCM or a close-up shot is frequently considered as a HT or short-period ST. Researchers have attempted to solve this problem by using BMA to compensate for previous or next frames. The similarity between compensated and current frames is computed to differentiate between OCM and transition. Usually, BMA is implemented with a small search area, such as 8×8, and small blocks, such as 8×8. This implementation is inefficient due to the high computational cost, and the motion is often greater than 8 pixels. Several researchers have attempted to tackle this problem by increasing the search area; however, such increase leads to an extremely high computational cost [164].

### 7.5. Changes in Small Regions (CSRs)

CSRs or picture-in-picture occurs in various video genres, such as news, movies, and advertisements. Transitions should be considered when a new locale is recorded by another camera. CSRs are regularly missed due to the low dissimilarities during transitions of small portions of frames [20].

Tackling CSRs requires the consideration of the following points: (1) utilizing local features in ROVI for multiple regions; and (2) detecting the transition for each region individually.

## 8. Unrevealed Issues and Future Direction

**Figure 12:**
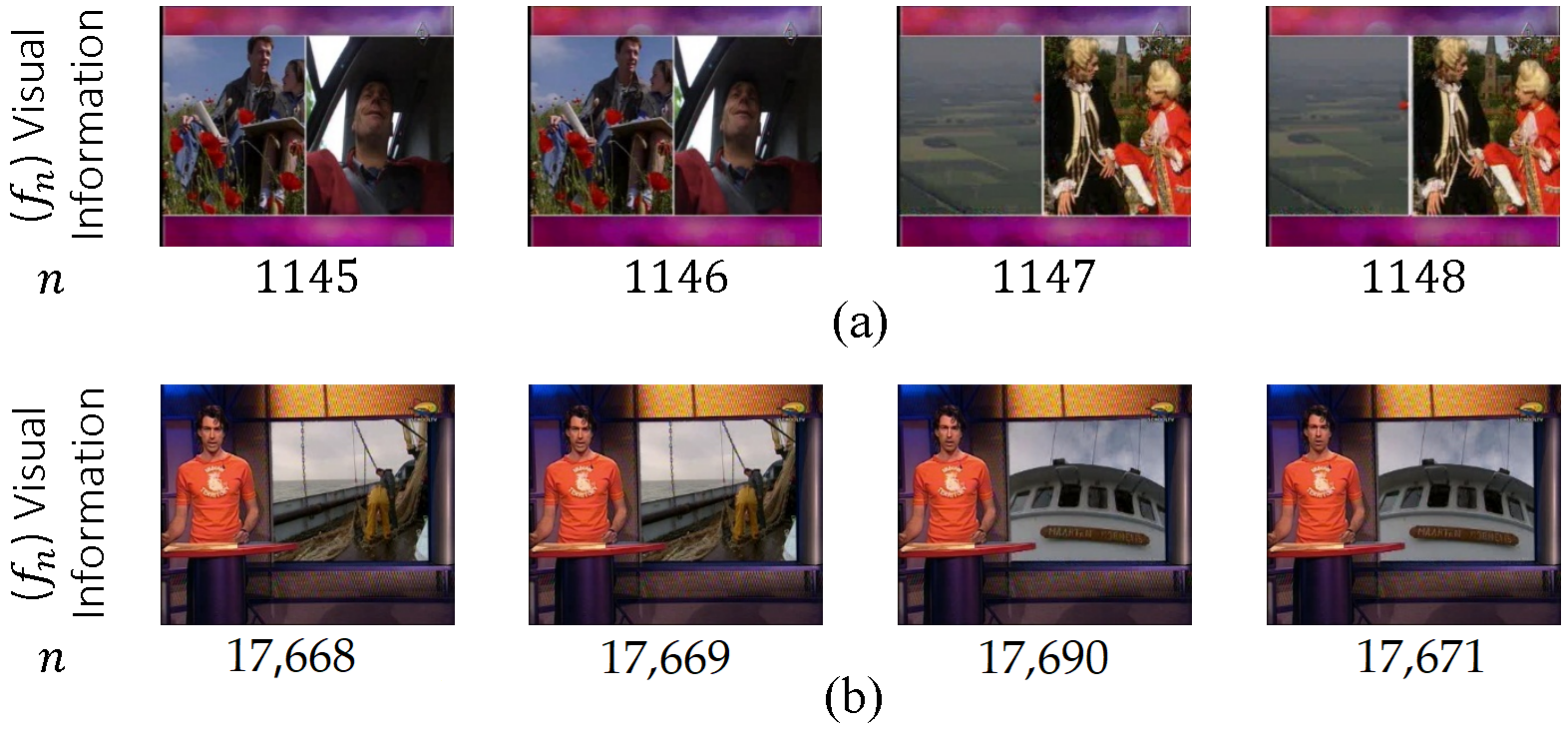
Examples of the CSRs extracted from TRECVID 2007: (**a**) Video ID: BG_2408; and (**b**) Video ID: BG_34901.

**Figure 13:**
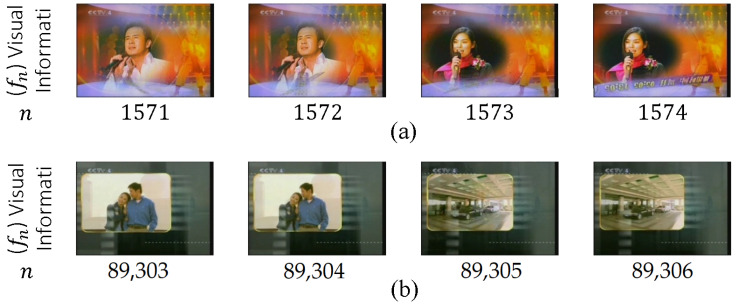
Examples of the CSRs extracted from TRECVID 2005, Video ID: 20041102_160001_CCTV4_DAILY_NEWS_CHN.

In this study, an attempt is made to highlight the current technologies in the field of SBD and to explore the advantages and disadvantages of each algorithm. However, it was difficult to establish an unbiased comparison among different algorithms because the results of the state-of-the-art studies were reported using different video datasets, performance analyses, and the experiments setups. Practically, most SBD studies either use subsets of standard datasets or use personal and unpublished datasets. Furthermore, most of the video datasets employed in the SBD do not cover the effects of all kinds of transitions. In this review, it was concluded that the TRECVID 2005, TRECVID 2006, and TRECVID 2007 datasets could be used all together in the future studies for performance evaluation. Although there are standard datasets, there are some conflicts between the ground truth and the actual type and location of the transitions. For instance, Figure 12 and Figure 13 show some examples of the CSRs that are not labeled in the ground truth source files, and this leads to make the SBD algorithm unable to evaluate the type of transition correctly. Here, an important question arises for discussion, should these unlabeled transitions be considered in evaluation? A comprehensive analysis is required to correct the ground truth of the corresponding videos as the wrong annotations lead to inaccurate and unstable machine-learning-based SBD algorithms.

In term of performance analysis, it is recommended that the researchers provide the details of the simulated results (predicted and true labels) in supplementary files which can be attached to the manuscript of their studies. In addition, it is useful to provide the performance scores (Precision, Recall, and F1-score) for each type of transition (fade in, fade out, fade out-in, dissolve, wipe, and other transitions) individually. As a result, the newcomers of video analysis will be able to track and duplicate the simulated results for the future studies.

In terms of performance analysis, reporting the achieved accuracies only is not enough to investigate the performance of the SBD, especially in the case of STs under different environments. In other words, the accuracies of the detected STs intervals are needed to be clearly reported.

Another problem is that some of the published studies did not provide the details of the hidden parameters to setup the experiments. For example, the SVM classifier is widely employed in SBD algorithms but the details on the SVM classifier parameters such as Gamma and cost functions are not clearly explained.

Many types of SBD algorithms have been successfully proposed by community to improve the performances and accuracies. Frame-skipping-based [15,38], Transform-based [125], and model-based [146] algorithms are good examples of the successful approaches. Recently, the machine learning algorithms received much attention in the field of computer vision applications. Exploring the benefit of the new machine learning technologies such as deep learning approaches for SBD could be directed as new directions for the future. In addition, proposing new types of features extraction, and dimensionality reduction methods could be useful to improve the performance of the current SBD algorithms.

## 9. Conclusions

Multimedia data, specifically video data, are available in huge volume. As a result, a demand for powerful indexing, retrieval, and summarization for multimedia data in cyberspace has appeared. Manual annotation and indexing for such sheer volume of data is inefficient. Thus, the need for automated and powerful video structure analysis are substantial. SBD is the first process in CBVIR, and its result influences the subsequent stages.

In this work, a comprehensive survey of SBD algorithms (or shot boundary detection algorithms) was performed. Video definitions, transition types, and hierarchies were demonstrated. The concepts of different basic and advanced SBD schemes were also presented. The details of the demonstrated algorithms were elaborated through the analysis of the modules, steps, merits, and demerits of SBD algorithms from different perspectives. The challenges in SBD were discussed, and several solutions were provided. The recommended datasets for evaluating SBD algorithms were also discussed, and evaluation metrics for SBD were provided.

It can be seen from this literature that researchers in the field of SBD have paid great efforts in developing algorithms for HTs and/or STs detection. These algorithms fall into two domains: compressed and uncompressed. Information in the compressed domain are used directly without the need for decoding process. The obscurity of the decoding process lead to fast algorithms; however, dependent on compression scheme. Hence, researchers shift toward features extracted from the uncompressed domain for the vast and valuable visual information. In the uncompressed domain, many algorithms have been presented to detect transitions. These algorithms are categorized into approaches based on the extracted features, which are: pixel-based, histogram-based, edge-based, transform-based, statistical-based, motion-based, and other. From the review, it can be seen that each approach has its own merits and demerits. Table 6 reported some of the state-of-the-art algorithms which are relied on several approaches. Although the performance of these algorithms is acceptable, an additional evaluation is required via standard, representative, and multiple challenging datasets (TRECVID 2005, 2006, and 2007). In addition, it can be observed that the accuracy and computational cost of the algorithms are contrastingly related, i.e., as the accuracy increase, the computational cost is increased and vice-versa. It is also noted that as the number of block size increased, the accuracy is increased on the account of computational complexity.

As concluded of the discussed results in this survey, SBD still have some problems that are relevant in practice for different video scenarios which need to be studied. These challenges are represented by: sudden illuminance change, dim lighting frames, comparable background frames, object and camera motion, and change in small regions. Solving these challenges will surely improve the performance of SBD algorithms.

## Figures and Tables

**Table 1:** Summary of PBA algorithms and their ability to detect transition type(s).

Ref	Color Space	Pre Processing	CDSS	CLDS Method			Post Processing	Transition Detection Ability			
				Threshold	Adaptive Threshold	SML		HT	Dissolve	Fade	Wipe
[68]	Gray&RGB	NA	City-Block	✔	-	-	NA	✔	-	-	-
[69]	Gray	Averaging Filter3x3	City-Block	✔	-	-	NA	✔	-	-	-
[70]	RGB	-combine 2 MSB from each space -Avg. Filter	City-Block	✔	-	-	NA	✔	✔	-	-
[24]	Gray	-Block Proc. (12 Block) -Block matching	NA	✔	-	-	NA	✔	✔	✔	-
[72]	RGB	NA	City-Block	-	✔	-	Flash Detector	✔	-	✔	-

**Table 2:** Summary of HBA algorithms and their ability to detect transition type(s).

Ref	Color Space	Pre Processing	Histogram Size	CDSS	CLDS Method			Post Processing	Transition Detection Ability			
					Threshold	Adaptive Threshold	SML		HT	Dissolve	Fade	Wipe
[69]	Gray	NA	NA	$\chi^{2}$	✔	-	-	NA	✔	-	-	-
[70]	Gray	NA	64	City-Block	✔	-	-	NA	✔	✔	-	-
[77]	RGB	NA	256	Weighted Difference	✔	-	-	NA	✔	-	-	-
[79]	Gray (global)	NA	64	City-Block	✔	-	-	NA	✔	-	-	-
[79]	Gray (16 region)	NA	64	City-Block	✔	-	-	NA	✔	-	-	-
[80]	RGB	RGB discretization	64 and 256	City-Block	✔	-	-	NA	✔	-	-	-
[81]	RGB	6-bit from each channel	64	Equation (15)	✔	-	-	NA	✔	-	-	-
[82]	RGB	6-bit from each channel	64	Equation (16)	✔	-	-	Temporal Skip	✔	-	-	-
[31]	Multiple Spaces	NA	256	Distances Spaces	✔	-	-	NA	✔	✔	-	-
[85]	RGB Global	NA	768	Equation (17)	-	-	GA	NA	✔	✔	✔	-
[86]	RGB	4-MSB from each space	4096	City-Block	✔	-	-	Refine Stage	✔	✔	✔	-
[87]	Gray	histogram quantized to 64-bins	64	Equation (18)	✔	-	-		-	-	✔	-
[88]	Gray	NA	NA	Equation (18)	✔	-	-		-	✔	-	-
[37]	L*a*b*	Video transformation detection	15	Fuzzy rules	✔	-	-		✔	✔	✔	-
[30]	RGB	NA	17,472	Histogram intersection	-	✔	-		✔	✔	✔	-
[90]	HSV + Gray	Resize frame	1024	City-Block	✔	-	-		✔	-	-	-
[91]	HSV	NA	NA	Euclidean	-	✔	-	-Gaussian filter -Voting mechanism	✔	✔	✔	-
[92]	HSV	frame resize	109	correlation	-	✔	-		✔	-	-	-
[94]	RGB	quantization to 8 levels	24	City-Block	✔	-	-	NA	✔	-	-	-

**Table 3:** Summary of EBA algorithms and their ability to detect transition type(s).

Ref	Color Space	Edge Operator	Pre Processing	CDSS	CLDS Method			Post Processing	Transition Detection Ability			
					Threshold	Adaptive Threshold	SML		HT	Dissolve	Fade	Wipe
[100]	Gray	Canny	Frame Smoothing	ECR	✔	-	-		✔	✔	✔	✔
[104]	Gray Ycbcr	wavelet	Temporal subsampling	Number of edge point	✔	-	-	refine stage	✔	✔	✔	-
[80]	Gray	Canny	NA	ECR	✔	-	-		✔	✔	✔	-
[106,107]	Gray and RGB or YUV	Canny	NA	Euclidean	✔	-	-	Refine stage	✔	-	✔	✔
[109]	Gray	Robert	NA	Number of edge pixels	✔	-	-		-	-	✔	-

**Table 4:** Summary of TBA algorithms and their ability to detect transition type(s).

Ref	Color Space	Transform	Pre Processing	CDSS	CLDS Method			Transition Detection Ability			
					Threshold	Adaptive Threshold	SML	HT	Dissolve	Fade	Wipe
[115]	Gray	DFT	Block 32 × 32	mean and std of normalized correlation	✔	-	-	✔	-	-	-
[116]	Luminance	DFT	Block processing	phase correlation	✔	-	-	✔	-	-	-
[117]	Gray	DFT	Block Processing	normalized correlation and median	✔	-	-	✔	✔	✔	-
[118]	Ohta Color Space	DCT	cosine similarity	NA	✔	-	-	✔	✔	-	-
[120]	Gray	DFT	spatial subsampling	Correlation	-	✔	-	✔	-	-	-
[121]	Gray	Walsh Hadamard	Frame resize	City-block	✔	-	-	✔	-	-	-

**Table 5:** Summary of SBA algorithms and their ability to detect transition type(s).

Ref	Color Space	Statistics	Pre Processing	CDSS	CLDS Method			Post Processing	Transition Detection Ability			
					Threshold	Adaptive Threshold	SML		HT	Dissolve	Fade	Wipe
[129]	Gray	Variance	NA	NA	✔	✔	-	NA	-	✔	-	-
[130]	Gray	mean and variance	NA	NA	-	✔	-	NA	-	✔	✔	-
[40]	DC images	likelihood function	NA	NA	✔	-	-	NA	✔	✔	-	-
[131]	RGB	mean standard deviation skew	NA	NA	✔	-	-	NA	✔	✔	✔	-

**Table 6:** Comparison of different state-of-the-art SBD algorithm.

Ref	Features	Frame Skipping	Dataset	P	R	*F*1	Computation Cost
[15]	Pixel Histogram	✔	2001 other	90.3	85.2	87.7	Low
[85]	Histogram	-	2001	88.7	93.1	90.7	Moderate
[33]	PBA CNN	✔	2001	91.2	84.2	87.5	Moderate
[57]	Gradient	-	2001 2007	90.5 87	87.6 89.9	89 88.4	Moderate
[125]	Walsh-Hadamard Motion	-	2001 2007	88.1 95.7	91.2 96.5	89.6 96.1	High
[1]	SURF	✔	2005	-	-	83	Moderate
[18]	Histogram Mutual Info Harris	✔	2001	93.5	94.5	93.99	Moderate
[41]	Histogram SIFT	✔	Other	96.6	93	94.7	Moderate
[138]	Histogram Fuzzy Color Histogram	-	Other	90.6	95.3	92.9	Moderate
[156]	SURF Histrogram	✔	2001	90.7	87.3	88.7	Moderate
[22]	Contourlet Transform 3 level of decomposition	-	2007	98	97	97.5	High

## References

[1] Birinci M., Kiranyaz S. (2014). A perceptual scheme for fully automatic video shot boundary detection. Signal Process. Image Commun..

[2] Gonzalez-Diaz I., Martinez-Cortes T., Gallardo-Antolin A., Diaz-de Maria F. (2015). Temporal segmentation and keyframe selection methods for user-generated video search-based annotation. Expert Syst. Appl..

[3] Priya R., Shanmugam T.N. (2013). A comprehensive review of significant researches on content based indexing and retrieval of visual information. Front. Comput. Sci..

[4] Yuan J., Wang H., Xiao L., Zheng W., Li J., Lin F., Zhang B. (2007). A formal study of shot boundary detection. IEEE Trans. Circ. Syst. Video Technol..

[5] Palmer S.E. (1999). Vision Science: Photons to Phenomenology.

[6] Del Fabro M., Böszörmenyi L. (2013). State-of-the-art and future challenges in video scene detection: A survey. Multimedia Syst..

[7] Fayk M.B., El Nemr H.A., Moussa M.M. (2010). Particle swarm optimisation based video abstraction. J. Adv. Res..

[8] Parmar M., Angelides M.C. (2015). MAC-REALM: A Video Content Feature Extraction and Modelling Framework. Comput. J..

[9] Hu W., Xie N., Li L., Zeng X., Maybank S. (2011). A Survey on Visual Content-Based Video Indexing and Retrieval. IEEE Trans. Syst. Man Cybern. Part C (Appl. Rev.).

[10] Choroś K., Hwang D., Jung J.J., Nguyen N.T. (2014). Improved Video Scene Detection Using Player Detection Methods in Temporally Aggregated TV Sports News. Proceedings of the 6th International Conference on Computational Collective Intelligence. Technologies and Applications, ICCCI 2014.

[11] Bhaumik H., Bhattacharyya S., Nath M.D., Chakraborty S. (2016). Hybrid soft computing approaches to content based video retrieval: A brief review. Appl. Soft Comput..

[12] Midya A., Sengupta S. (2015). Switchable video error concealment using encoder driven scene transition detection and edge preserving SEC. Multimedia Tools Appl..

[13] Liu T., Kender J.R. (2007). Computational approaches to temporal sampling of video sequences. ACM Trans. Multimedia Comput. Commun. Appl. (TOMM).

[14] Trichet R., Nevatia R., Burns B. Video event classification with temporal partitioning. Proceedings of the 2015 12th IEEE International Conference on Advanced Video and Signal Based Surveillance (AVSS).

[15] Lu Z.M., Shi Y. (2013). Fast video shot boundary detection based on SVD and pattern matching. IEEE Trans. Image Process..

[16] Liu C., Wang D., Zhu J., Zhang B. (2013). Learning a Contextual Multi-Thread Model for Movie/TV Scene Segmentation. IEEE Trans Multimedia.

[17] Tavassolipour M., Karimian M., Kasaei S. (2014). Event Detection and Summarization in Soccer Videos Using Bayesian Network and Copula. IEEE Trans. Circ. Syst. Video Technol..

[18] Gao G., Ma H. (2014). To accelerate shot boundary detection by reducing detection region and scope. Multimedia Tools Appl..

[19] Pal G., Rudrapaul D., Acharjee S., Ray R., Chakraborty S., Dey N., Satapathy C.S., Govardhan A., Raju S.K., Mandal K.J. (2015). Video Shot Boundary Detection: A Review. Emerging ICT for Bridging the Future—Proceedings of the 49th Annual Convention of the Computer Society of India CSI Volume 2.

[20] Choroś K., Zgrzywa A., Choroś K., Siemiński A. (2015). False and miss detections in temporal segmentation of TV sports news videos–causes and remedies. New Research in Multimedia and Internet Systems.

[21] Iwan L.H., Thom J.A. (2017). Temporal video segmentation: detecting the end-of-act in circus performance videos. Multimedia Tools Appl..

[22] Mondal J., Kundu M.K., Das S., Chowdhury M. (2017). Video shot boundary detection using multiscale geometric analysis of nsct and least squares support vector machine. Multimedia Tools Appl..

[23] Dutta D., Saha S.K., Chanda B. (2016). A shot detection technique using linear regression of shot transition pattern. Multimedia Tools Appl..

[24] Shahraray B. (1995). Scene change detection and content-based sampling of video sequences. IS&T/SPIE’s Symposium on Electronic Imaging: Science & Technology.

[25] Kar T., Kanungo P. (2017). A motion and illumination resilient framework for automatic shot boundary detection. Signal Image Video Process.

[26] Duan L.Y., Xu M., Tian Q., Xu C.S., Jin J.S. (2005). A unified framework for semantic shot classification in sports video. IEEE Trans. Multimedia.

[27] Amiri A., Fathy M. (2010). Video shot boundary detection using QR-decomposition and gaussian transition detection. EURASIP J. Adv. Signal Process..

[28] Ren W., Sharma M. Automated video segmentation. Proceedings of the 3rd International Conference on Information, Communication, and Signal Processing.

[29] Xiong W., Lee C.M., Ma R.H. (1997). Automatic video data structuring through shot partitioning and key-frame computing. Mach. Vis. Appl..

[30] Janwe N.J., Bhoyar K.K. Video shot boundary detection based on JND color histogram. Proceedings of the 2013 IEEE Second International Conference on Image Information Processing (ICIIP).

[31] Gargi U., Kasturi R., Strayer S.H. (2000). Performance Characterization of Video-Shot-Change Detection Methods. IEEE Trans. Circ. Syst..

[32] Chen Y., Deng Y., Guo Y., Wang W., Zou Y., Wang K. A Temporal Video Segmentation and Summary Generation Method Based on Shots’ Abrupt and Gradual Transition Boundary Detecting. Proceedings of the 2010 ICCSN’10 Second International Conference on Communication Software and Networks.

[33] Tong W., Song L., Yang X., Qu H., Xie R. CNN-Based Shot Boundary Detection and Video Annotation. Proceedings of the 2015 IEEE International Symposium on Broadband Multimedia Systems and Broadcasting.

[34] Asghar M.N., Hussain F., Manton R. (2014). Video indexing: A survey. Int. J. Comput. Inf. Technol..

[35] Kowdle A., Chen T., Fitzgibbon A., Lazebnik S., Perona P., Sato Y., Schmid C. (2012). Learning to Segment a Video to Clips Based on Scene and Camera Motion. Proceedings of the Computer Vision—ECCV 2012: 12th European Conference on Computer Vision.

[36] Bescós J., Cisneros G., Martínez J.M., Menéndez J.M., Cabrera J. (2005). A unified model for techniques on video-shot transition detection. IEEE Trans. Multimedia.

[37] Küçüktunç O., Güdükbay U., Ulusoy Ö. (2010). Fuzzy color histogram-based video segmentation. Comput. Vis. Image Underst..

[38] Li Y.N., Lu Z.M., Niu X.M. (2009). Fast video shot boundary detection framework employing pre-processing techniques. IET Image Process..

[39] BABER J., Afzulpurkar N., Satoh S. (2013). A framework for video segmentation using global and local features. Int. J. Pattern Recognit. Art. Intell..

[40] Hanjalic A. (2002). Shot-boundary detection: Unraveled and resolved?. IEEE Trans. Circ. Syst. Video Technol..

[41] Jiang X., Sun T., Liu J., Chao J., Zhang W. (2013). An adaptive video shot segmentation scheme based on dual-detection model. Neurocomputing.

[42] Cao J., Cai A. (2007). A robust shot transition detection method based on support vector machine in compressed domain. Pattern Recognit. Lett..

[43] Hampapur A., Jain R., Weymouth T.E. (1995). Production model based digital video segmentation. Multimedia Tools Appl..

[44] Ling X., Yuanxin O., Huan L., Zhang X. A Method for Fast Shot Boundary Detection Based on SVM. Proceedings of the 2008 CISP’08 Congress on Image and Signal Processing.

[45] Fang H., Jiang J., Feng Y. (2006). A fuzzy logic approach for detection of video shot boundaries. Pattern Recognit..

[46] Choroś K., Gonet M. (2008). Effectiveness of video segmentation techniques for different categories of videos. New Trends Multimedia Netw. Inf. Syst..

[47] Choroś K., Nguyen N.T. (2011). Reduction of faulty detected shot cuts and cross dissolve effects in video segmentation process of different categories of digital videos. Transactions on Computational Collective Intelligence V.

[48] Černeková Z., Kotropoulos C., Pitas I. (2007). Video shot-boundary detection using singular-value decomposition and statistical tests. J. Electron. Imaging.

[49] Joyce R.A., Liu B. (2006). Temporal segmentation of video using frame and histogram space. IEEE Trans. Multimedia.

[50] Černeková Z., Pitas I., Nikou C. (2006). Information theory-based shot cut/fade detection and video summarization. IEEE Trans. Circ. Syst. Video Technol..

[51] Porter S.V. (2004). Video Segmentation and Indexing Using Motion Estimation. Ph.D. Thesis.

[52] Barbu T. (2009). Novel automatic video cut detection technique using Gabor filtering. Comput. Electr. Eng..

[53] Zheng W., Yuan J., Wang H., Lin F., Zhang B. (2005). A novel shot boundary detection framework. Visual Communications and Image Processing 2005.

[54] Kawai Y., Sumiyoshi H., Yagi N. (2007). Shot boundary detection at TRECVID 2007. Proceedings of the TRECVID 2007 Workshop.

[55] Hameed A. A novel framework of shot boundary detection for uncompressed videos. Proceedings of the 2009 ICET 2009 International Conference on Emerging Technologies.

[56] Cotsaces C., Nikolaidis N., Pitas I. (2006). Video shot boundary detection and condensed representation: A review. IEEE Signal Process. Mag..

[57] Shekar B.H., Uma K.P. (2015). Kirsch Directional Derivatives Based Shot Boundary Detection: An Efficient and Accurate Method. Procedia Comput. Sci..

[58] Amiri A., Fathy M. (2011). Video Shot Boundary Detection Using Generalized Eigenvalue Decomposition And Gaussian Transition Detection. Comput. Inform..

[59] Over P., Ianeva T., Kraaij W., Smeaton A.F., Val U.D. (2005). TRECVID 2005—An Overview.

[60] Lefèvre S., Vincent N. (2007). Efficient and robust shot change detection. J. Real-Time Image Process..

[61] Cooper M., Liu T., Rieffel E. (2007). Video Segmentation via Temporal Pattern Classification. IEEE Trans. Multimedia.

[62] Grana C., Cucchiara R. (2007). Linear transition detection as a unified shot detection approach. IEEE Trans. Circ. Syst. Video Technol..

[63] Aryal S., Ting K.M., Washio T., Haffari G. (2017). Data-dependent dissimilarity measure: An effective alternative to geometric distance measures. Knowl. Inf. Syst..

[64] Le D.D., Satoh S., Ngo T.D., Duong D.A. A text segmentation based approach to video shot boundary detection. Proceedings of the 2008 IEEE 10th Workshop on Multimedia Signal Processing.

[65] Camara-Chavez G., Precioso F., Cord M., Phillip-Foliguet S., de A. Araujo A. Shot Boundary Detection by a Hierarchical Supervised Approach. Proceedings of the 14th International Workshop on Systems, Signals and Image Processing and 6th EURASIP Conference focused on Speech and Image Processing, Multimedia Communications and Services.

[66] Pacheco F., Cerrada M., Sánchez R.V., Cabrera D., Li C., de Oliveira J.V. (2017). Attribute clustering using rough set theory for feature selection in fault severity classification of rotating machinery. Expert Syst. Appl..

[67] Lee M.S., Yang Y.M., Lee S.W. (2001). Automatic video parsing using shot boundary detection and camera operation analysis. Pattern Recognit..

[68] Kikukawa T., Kawafuchi S. (1992). Development of an automatic summary editing system for the audio-visual resources. Trans. Inst. Electron. Inf. Commun. Eng..

[69] Nagasaka A., Tanaka Y. (1992). Automatic video indexing and full-video search for object appearances. Visual Database Systems II.

[70] Zhang H., Kankanhalli A., Smoliar S.W. (1993). Automatic partitioning of full-motion video. Multimedia Syst..

[71] Lian S. (2011). Automatic video temporal segmentation based on multiple features. Soft Comput..

[72] Yeo B.L., Liu B. (1995). Rapid Scene Analysis on Compressed Video. IEEE Trans. Circ. Syst. Video Technol..

[73] Huan Z.H.Z., Xiuhuan L.X.L., Lilei Y.L.Y. (2008). Shot Boundary Detection Based on Mutual Information and Canny Edge Detector. 2008 Int. Conf. Comput. Sci. Softw. Engineering.

[74] Koprinska I., Carrato S. (2001). Temporal video segmentation: A survey. Signal Process. Image Commun..

[75] Tapu R., Zaharia T. (2011). Video Segmentation and Structuring for Indexing Applications. Int. J. Multimedia Data Eng. Manag..

[76] Ciocca G., Schettini R. Dynamic storyboards for video content summarization. Proceedings of the MIR’06 8th ACM International Workshop on Multimedia Information Retrieval.

[77] Swanberg D., Shu C.F., Jain R.C. (1993). Knowledge-guided parsing in video databases. IS&T/SPIE’s Symposium on Electronic Imaging: Science and Technology.

[78] Solomon C., Breckon T. (2011). Fundamentals of Digital Image Processing: A Practical Approach with Examples in Matlab.

[79] Boreczky J.S., Rowe L.a. (1996). Comparison of video shot boundary detection techniques. J. Electron. Imaging.

[80] Lienhart R.W. (1998). Comparison of automatic shot boundary detection algorithms. Proceedings of SPIE Storage and Retrieval for Image and Video Databases VII;.

[81] Ahmed M., Karmouch A., Abu-Hakima S. Key Frame Extraction and Indexing for Multimedia Databases. Proceedings of the Vision Interface’99.

[82] Ahmed M., Karmouch A. Video segmentation using an opportunistic approach. Proceedings of the International Conference on Multimedia Modeling 1999.

[83] Shih T.Y. (1995). The reversibility of six geometric color spaces. Photogramm. Eng. Remote Sens..

[84] Tkalcic M., Tasic J.F. Colour spaces: Perceptual, historical and applicational background. Proceedings of the IEEE Region 8, EUROCON 2003, Computer as a Tool.

[85] Thounaojam D.M., Khelchandra T., Singh K.M., Roy S. (2016). A Genetic Algorithm and Fuzzy Logic Approach for Video Shot Boundary Detection. Comput. Intell. Neurosci..

[86] Mas J., Fernandez G. (2003). Video shot boundary detection based on color histogram. Notebook Papers TRECVID 2003.

[87] Qian X., Liu G., Su R. (2006). Effective Fades and Flashlight Detection Based on Accumulating Histogram Difference. IEEE Trans. Circ. Syst. Video Technol..

[88] Ji Q.G., Feng J.W., Zhao J., Lu Z.M. Effective Dissolve Detection Based on Accumulating Histogram Difference and the Support Point. Proceedings of the 2010 First International Conference on Pervasive Computing Signal Processing and Applications (PCSPA).

[89] Bhoyar K., Kakde O. (2010). Color image segmentation based on JND color histogram. Int. J. Image Process. (IJIP).

[90] Adnan A., Ali M. (2013). Shot boundary detection using sorted color histogram polynomial curve. Life Sci. J..

[91] Li Z., Liu X., Zhang S. Shot Boundary Detection based on Multilevel Difference of Colour Histograms. Proceedings of the 2016 First International Conference on Multimedia and Image Processing (ICMIP), Bandar Seri Begawan.

[92] Park S., Son J., Kim S.J. Effect of adaptive thresholding on shot boundary detection performance. Proceedings of the 2016 IEEE International Conference on Consumer Electronics-Asia (ICCE-Asia).

[93] Park S., Son J., Kim S.J. Study on the effect of frame size and color histogram bins on the shot boundary detection performance. Proceedings of the 2016 IEEE International Conference on Consumer Electronics-Asia (ICCE-Asia).

[94] Verma M., Raman B., Raman B., Kumar S., Roy P.P., Sen D. (2017). A Hierarchical Shot Boundary Detection Algorithm Using Global and Local Features. Proceedings of International Conference on Computer Vision and Image Processing: CVIP 2016, Volume 2.

[95] Pye D., Hollinghurst N.J., Mills T.J., Wood K.R. Audio-visual segmentation for content-based retrieval. Proceedings of the 5th International Conference on Spoken Language Processing (ICSLP’98).

[96] Dailianas A., Allen R.B., England P. (1996). Comparison of automatic video segmentation algorithms. Proceedings of SPIE–The International Society for Optical Engineering;.

[97] Lienhart R.W. (2001). Reliable transition detection in videos: A survey and practitioner’s guide. Int. J. Image Graph..

[98] Heng W.J., Ngan K.N. (2003). High accuracy flashlight scene determination for shot boundary detection. Signal Process. Image Commun..

[99] Kim S.H., Park R.H. Robust video indexing for video sequences with complex brightness variations. Proceedings of the lnternational Conference on Signal and Image Processing.

[100] Zabih R., Miller J., Mai K. A Feature-Based Algorithm for Detecting and Classifying Scene Breaks. Proceedings of the Third ACM International Conference on Multimedia Multimedia 95;.

[101] Canny J. (1986). A computational approach to edge detection. IEEE Trans. Pattern Anal. Mach. Intell..

[102] Zabih R., Miller J., Mai K. (1999). A feature-based algorithm for detecting and classifying production effects. Multimedia Syst..

[103] Lupatini G., Saraceno C., Leonardi R. Scene break detection: a comparison. Proceedings of the Eighth IEEE International Workshop on Research Issues In Data Engineering, ‘Continuous-Media Databases and Applications’.

[104] Nam J., Tewfik A.H. Combined audio and visual streams analysis for video sequence segmentation. Proceedings of the 1997 IEEE International Conference on Acoustics, Speech, and Signal Processing, (ICASSP-97).

[105] Lienhart R.W. (2001). Reliable dissolve detection. Photonics West 2001-Electronic Imaging.

[106] Heng W.J., Ngan K.N. Integrated shot boundary detection using object-based technique. Proceedings of the 1999 IEEE International Conference on Image Processing, 1999 ICIP 99.

[107] Heng W.J., Ngan K.N. (2001). An Object-Based Shot Boundary Detection Using Edge Tracing and Tracking. J. Vis. Commun. Image Represent..

[108] Roberts L.G. (1963). Machine Perception of Three-Dimensional Soups. Ph.D. Thesis.

[109] Zheng J., Zou F., Shi M. An efficient algorithm for video shot boundary detection. Proceedings of the 2004 IEEE International Symposium on Intelligent Multimedia, Video and Speech Processing.

[110] Mahmmod B.M., Ramli A.R., Abdulhussain S.H., Al-Haddad S.A.R., Jassim W.A. (2017). Low-Distortion MMSE Speech Enhancement Estimator Based on Laplacian Prior. IEEE Access.

[111] ABDULHUSSAIN S.H., Ramli A.R., Mahmmod B.M., Al-Haddad S.A.R., Jassim W.A. (2017). Image Edge Detection Operators based on Orthogonal Polynomials. Int. J. Image Data Fusion.

[112] Abdulhussain S.H., Ramli A.R., Al-Haddad S.A.R., Mahmmod B.M., Jassim W.A. (2017). On Computational Aspects of Tchebichef Polynomials for Higher Polynomial Order. IEEE Access.

[113] Mahmmod B.M., Ramli A.R., Abdulhussain S.H., Al-Haddad S.A.R., Jassim W.A. (2018). Signal Compression and Enhancement Using a New Orthogonal-Polynomial-Based Discrete Transform. IET Signal Process..

[114] Abdulhussain S.H., Ramli A.R., Al-Haddad S.A.R., Mahmmod B.M., Jassim W.A. (2018). Fast Recursive Computation of Krawtchouk Polynomials. J. Math. Imaging Vis..

[115] Porter S.V., Mirmehdi M., Thomas B.T. Video cut detection using frequency domain correlation. Proceedings of the IEEE 15th International Conference on Pattern Recognition.

[116] Vlachos T. (2000). Cut detection in video sequences using phase correlation. IEEE Signal Process. Lett..

[117] Porter S., Mirmehdi M., Thomas B. (2003). Temporal video segmentation and classification of edit effects. Image Vis. Comput..

[118] Cooper M., Foote J., Adcock J., Casi S. (2003). Shot boundary detection via similarity analysis. Proceedings of the TRECVID Workshop.

[119] Ohta Y.I., Kanade T., Sakai T. (1980). Color information for region segmentation. Comput. Graph. Image Process..

[120] Urhan O., Gullu M.K., Erturk S. (2006). Modified phase-correlation based robust hard-cut detection with application to archive film. IEEE Trans. Circ. Syst. Video Technol..

[121] Priya G.L., Domnic S. (2012). Edge Strength Extraction using Orthogonal Vectors for Shot Boundary Detection. Procedia Technol..

[122] Bouthemy P., Gelgon M., Ganansia F. (1999). A unified approach to shot change detection and camera motion characterization. IEEE Trans. Circ. Syst. Video Technol..

[123] Dufaux F., Konrad J. (2000). Efficient, robust, and fast global motion estimation for video coding. IEEE Trans. Image Process..

[124] Bruno E., Pellerin D. Video shot detection based on linear prediction of motion. Proceedings of the 2002 IEEE International Conference on Multimedia and Expo (ICME’02).

[125] Priya L.G.G., Domnic S. (2014). Walsh—Hadamard Transform Kernel-Based Feature Vector for Shot Boundary Detection. IEEE Trans. Image Process..

[126] Barjatya A. (2004). Block matching algorithms for motion estimation. IEEE Trans. Evol. Comput..

[127] Zedan I.A., Elsayed K.M., Emary E., Hassanien A.E., Shaalan K., Gaber T., Azar A.T., Tolba M.F. (2017). Abrupt Cut Detection in News Videos Using Dominant Colors Representation. Proceedings of the International Conference on Advanced Intelligent Systems and Informatics 2016;.

[128] Jain R., Kasturi R. (1991). Dynamic vision. Computer Vision: Principles.

[129] Alattar A.M. Detecting And Compressing Dissolve Regions In Video Sequences With A DVI Multimedia Image Compression Algorithm. Proceedings of the 1993 IEEE International Symposium on Circuits and Systems (ISCAS’93).

[130] Truong B.T., Dorai C., Venkatesh S. (2000). New Enhancements to Cut, Fade, and Dissolve Detection Processes in Video Segmentation. Proceedings of the Eighth ACM International Conference on Multimedia.

[131] Miadowicz J.Z. (2004). Story Tracking in Video News Broadcasts. Ph.D. Thesis.

[132] Ribnick E., Atev S., Masoud O., Papanikolopoulos N., Voyles R. Real-time detection of camera tampering. Proceedings of the IEEE International Conference on Video and Signal Based Surveillance (AVSS’06).

[133] Chung M.G., Kim H., Song S.M.H. A scene boundary detection method. Proceedings of the 2000 International Conference on Image Processing.

[134] Ngo C.W., Pong T.C., Chin R.T. (2001). Video partitioning by temporal slice coherency. IEEE Trans. Circ. Syst. Video Technol..

[135] Ferman A.M., Tekalp A.M. (1998). Efficient filtering and clustering methods for temporal video segmentation and visual summarization. J. Vis. Commun. Image Represent..

[136] Duda R.O., Hart P.E. (1972). Use of the Hough transformation to detect lines and curves in pictures. Commun. ACM.

[137] Meng J., Juan Y., Chang S.F. Scene Change Detection in a MPEG compressed Video Sequence. Proceedings of the IS&T/SPIE International Symposium on Electronic Imaging, Science & Technology.

[138] Dadashi R., Kanan H.R. (2013). AVCD-FRA: A novel solution to automatic video cut detection using fuzzy-rule-based approach. Comput. Vis. Image Underst..

[139] Nishani E., Çiço B. Computer vision approaches based on deep learning and neural networks: Deep neural networks for video analysis of human pose estimation. Proceedings of the 2017 6th Mediterranean Conference on Embedded Computing (MECO).

[140] Xu J., Song L., Xie R. Shot boundary detection using convolutional neural networks. Proceedings of the 2016 Visual Communications and Image Processing (VCIP).

[141] Birinci M., Kiranyaz S., Gabbouj M. Video shot boundary detection by structural analysis of local image features. Proceedings of the WIAMIS 2011: 12th International Workshop on Image Analysis for Multimedia Interactive Services.

[142] Bay H., Tuytelaars T., Van Gool L. (2006). SURF: Speeded up robust features. European Conference on Computer Vision.

[143] Bhaumik H., Chakraborty M., Bhattacharyya S., Chakraborty S. (2017). Detection of Gradual Transition in Videos: Approaches and Applications. Intelligent Analysis of Multimedia Information.

[144] Chan C., Wong A. Shot Boundary Detection Using Genetic Algorithm Optimization. Proceedings of the 2011 IEEE International Symposium on Multimedia (ISM).

[145] Jaffré G., Joly P., Haidar S. (2004). The Samova Shot Boundary Detection for TRECVID Evaluation 2004.

[146] Mohanta P.P., Saha S.K., Chanda B. (2012). A model-based shot boundary detection technique using frame transition parameters. IEEE Trans. Multimedia.

[147] Lankinen J., Kämäräinen J.K. Video Shot Boundary Detection using Visual Bag-of-Words. Proceedings of the VISAPP 2013—Proceedings of the International Conference on Computer Vision Theory and Applications.

[148] ImageNet. http://www.image-net.org/.

[149] Bhalotra P.S.A., Patil B.D. (2013). Shot boundary detection using radon projection method. Int. J. Signal Image Process..

[150] Miene A., Hermes T., Ioannidis G.T., Herzog O. (2003). Automatic shot boundary detection using adaptive thresholds. Proceedings of the TRECVID 2003 Workshop.

[151] Chen J., Ren J., Jiang J. (2011). Modelling of content-aware indicators for effective determination of shot boundaries in compressed MPEG videos. Multimedia Tools Appl..

[152] Guimaraes S.J.F., do Patrocinio Z.K.G., Souza K.J.F., de Paula H.B. Gradual transition detection based on bipartite graph matching approach. Proceedings of the IEEE International Workshop on Multimedia Signal Processing (MMSP’09).

[153] Yoo H.W., Ryoo H.J., Jang D.S. (2006). Gradual shot boundary detection using localized edge blocks. Multimedia Tools Appl..

[154] Sobel I., Feldman G. (1968). A 3 × 3 isotropic gradient operator for image processing. Presented at a Talk at the Stanford Artificial Project. Pattern Classification and Scene Analysis.

[155] Hall M., Frank E., Holmes G., Pfahringer B., Reutemann P., Witten I.H. (2009). The WEKA data mining software: an update. ACM SIGKDD Explor. Newslett..

[156] Tippaya S., Sitjongsataporn S., Tan T., Khan M.M., Chamnongthai K. (2017). Multi-Modal Visual Features-Based Video Shot Boundary Detection. IEEE Access.

[157] Ahmed A. (2009). Video Representation and Processing for Multimedia Data Mining. Semantic Mining Technologies for Multimedia Databases.

[158] Van Rijsbergen C.J. (1979). Information Retrieval.

[159] Makhoul J., Kubala F., Schwartz R., Weischedel R. Performance measures for information extraction. Proceedings of the DARPA Broadcast News Workshop.

[160] Lefevre S., Holler J., Vincent N. (2003). A review of real-time segmentation of uncompressed video sequences for content-based search and retrieval. Real-Time Imaging.

[161] TRECVID. http://trecvid.nist.gov.

[162] Chen L.H., Hsu B.C., Su C.W. (2017). A Supervised Learning Approach to Flashlight Detection. Cybern. Syst..

[163] Parnami N.M.N.S.A., Chandran S.L.S. (2006). Indian Institute of Technology, Bombay at TRECVID 2006. Proceedings of the TRECVID Workshop.

[164] Mishra R., Singhai S.K., Sharma M. Comparative Study of Block Matching Algorithm and Dual Tree Complex Wavelet Transform for Shot Detection in Videos. Proceedings of the 2014 International Conference on Electronic Systems, Signal Processing and Computing Technologies (ICESC).

